# A Novel Survey for Assessing Health‐Promoting School Practices and Capacity: Validation and Adaptability to Physical Activity

**DOI:** 10.1111/josh.70071

**Published:** 2025-09-04

**Authors:** Anneke Vang Hjort, Adrian Bauman, Sofie Rolff Carlsen, Johanne Triantafyllou Lorenzen, Charlotte Demant Klinker

**Affiliations:** ^1^ Centre for Childhood Health Copenhagen Denmark; ^2^ Systems Approaches in Public Health Copenhagen University Hospital – Steno Diabetes Center Copenhagen Herlev Denmark; ^3^ School of Public Health Sydney University Sydney Australia

**Keywords:** health‐promoting school, implementation assessment, measurement tools, physical activity, survey validation, whole school approach

## Abstract

**Background:**

Understanding current practice is crucial for implementing Health Promoting Schools (HPS) and Whole School Approaches to Physical Activity (WSA‐PA). This study validated two survey tools to assess HPS and WSA‐PA implementation, respectively.

**Methods:**

Content validity was established via literature review and expert and target group consultations. Teachers at Danish schools for vulnerable youth (ages 16–25) completed surveys in fall 2023. Structural validity was assessed using confirmatory factor analysis, construct validity via the Multitrait‐Multimethod (MTMM) approach, and reliability with Cronbach's Alpha, Omega H, and Intraclass Correlation Coefficient.

**Results:**

A total of 921 staff at 78 schools participated (72% response rate). The HPS scale had 24 items, while the WSA‐PA had 21 across various subfactors, such as school ethos, health services, and resources. Statistical analyses demonstrated good structural validity (e.g., RMSEA = 0.023 for HPS, RMSEA = 0.043 for WSA‐PA) and acceptable reliability. MTMM confirmed both convergent and discriminant validity.

**Implications for School Health Policy, Practice, and Equity:**

These tools can help schools monitor and improve HPS and WSA‐PA implementation, supporting equity and informing national policies.

**Conclusion:**

The validated tools can guide schools in enhancing HPS and WSA‐PA practices. Further research should refine these instruments.

## 1 Introduction

The concept of Health Promoting School (HPS) is a whole‐school approach that capitalizes on the organizational capacity of schools to create and maintain conditions conducive to both health and educational outcomes for students [1, 2, 3, 4, 5]. The HPS extends beyond curricular activity or isolated health promoting programs to encompass an overall school ethos and both the physical and psychosocial environments [1, 4]. Components of an HPS include distributed governance, which encourages the active involvement of students, staff, and the broader community in health promotion planning and implementation [1, 2]. Additionally, the HPS framework underscores the importance of school policies that embed health promotion and prevention within the school's rules, alongside continuous monitoring of implementation and impact [1, 2, 3, 4]. Furthermore, the HPS aims to foster action competencies among students and facilitate access to healthcare services [1, 4, 6].

The HPS was introduced by the World Health Organization (WHO) in the late 1980s and has shown positive outcomes concerning physical activity, physical fitness, body mass index (BMI), and fruit and vegetable intake [7], improved social and emotional skills among students [8], and might increase mental health and job commitment among teaching staff [9]. Hence, the HPS is widely accepted as an effective strategy to promote health and well‐being.

A Whole‐School Approach to Physical Activity (WSA‐PA) is based on the broad principles of the HPS framework applied specifically to physical activity. This comprises school policies on physical activity, developing action competencies related to physical activity, collaborating with local physical activity partners, and promoting active transportation to and from school [10, 11].

Despite growing global adoption [12, 13, 14, 15], many schools struggle with implementing the HPS or similar whole‐school approaches [16]. This is critical, as the success of any program is dependent on how well it is implemented [17]. Hence, inconsistencies in the effectiveness of HPS and WSA‐PA may be attributed to inadequate implementation [18, 19]. Given the comprehensive nature of the HPS framework, it is likely that schools face greater challenges in implementing certain aspects of the framework compared to others. Consequently, a first step is monitoring HPS and WSA‐PA practices and capacity. This can guide schools in strengthening their implementation strategies in areas with low capacity as well as guide policy and practice at the national level.

Many surveys have been developed to assess the HPS [10, 20, 21, 22, 23, 24, 25, 26, 27]. The WHO [28] and the US Centers for Disease Prevention and Control (CDC) [29] also offer surveys to monitor school health policies and practices. However, most school health surveys are not psychometrically tested [30]. Moreover, we note that most HPS surveys are not distinctly operationalized; that is, the surveys [10, 20, 21, 22, 23, 24, 25, 26, 27] rely on (broad) definitions of HPS—but do not clarify which aspects of HPS they aim to capture or how they attempt to measure them. This makes it difficult to appraise and adapt previous HPS surveys.

Specifically, most prior surveys include items on the overall HPS framework alongside items on specific health behaviors, often focusing on physical activity and diet [10, 20, 24, 30]. An exception is a recent Dutch HPS survey, which covers various behaviors, such as nutrition, well‐being, substance abuse, media literacy, and sexuality [21]. Nonetheless, previous surveys generally lack clarity on how they operationalize general HPS versus specific health behaviors and why they mainly focus on physical activity and diet. To our knowledge, no survey has comprehensively captured core HPS indicators while offering transparent flexibility to tailor the survey to specific health behaviors.

## 2 Aim

This study aimed to develop and validate two survey instruments: one that captures overall HPS practices and capacity, and another that specifically assesses WSA‐PA. The rationale behind this approach is to create one survey that captures the core indicators of the HPS and a second survey that illustrates how these core indicators can be adapted to address a whole school approach to specific health behaviors, in this case, physical activity.

To ensure utility for both practice and research, the secondary aims were to (1) explain the operationalization to enable other researchers to build upon the tools and (2) develop guidelines that facilitate the practical usability of the tools.

## 3 Methods

### 3.1 Setting

This survey validation study was conducted at Danish *Preparatory Basic Education‐ and Training* (Danish: FGU) schools. The schools target youth between the ages of 15 and 25 (mean: 19 years) who are “Not in Education, Employment, or Training” (NEET) and hence need extra support to enter the conventional education system or workforce. The length of the FGU school program depends on individual needs but typically does not exceed 2 years, and it includes additional vocational, social, or personal support and skill development. In 2023, the total FGU student population was approximately *n* = 9000 across 27 FGU institutions covering 85 school premises (physical addresses).

### 3.2 Instrumentation, Procedures, and Data Analysis

The study is informed by the two complementary guidelines for the development and validation of survey tools: the *COSMIN Risk of Bias Checklist for Patient‐Reported Outcome Measures* [31] and the *Primer for Best Practices in Developing and Validating Scales for Health, Social, and Behavioral Research* [32]. Supporting Information: Appendix 1 provides a comprehensive list and description of these guidelines, along with an explanation of how the study aligns with them, aiding readers in critically appraising this research.

**FIGURE 1:**
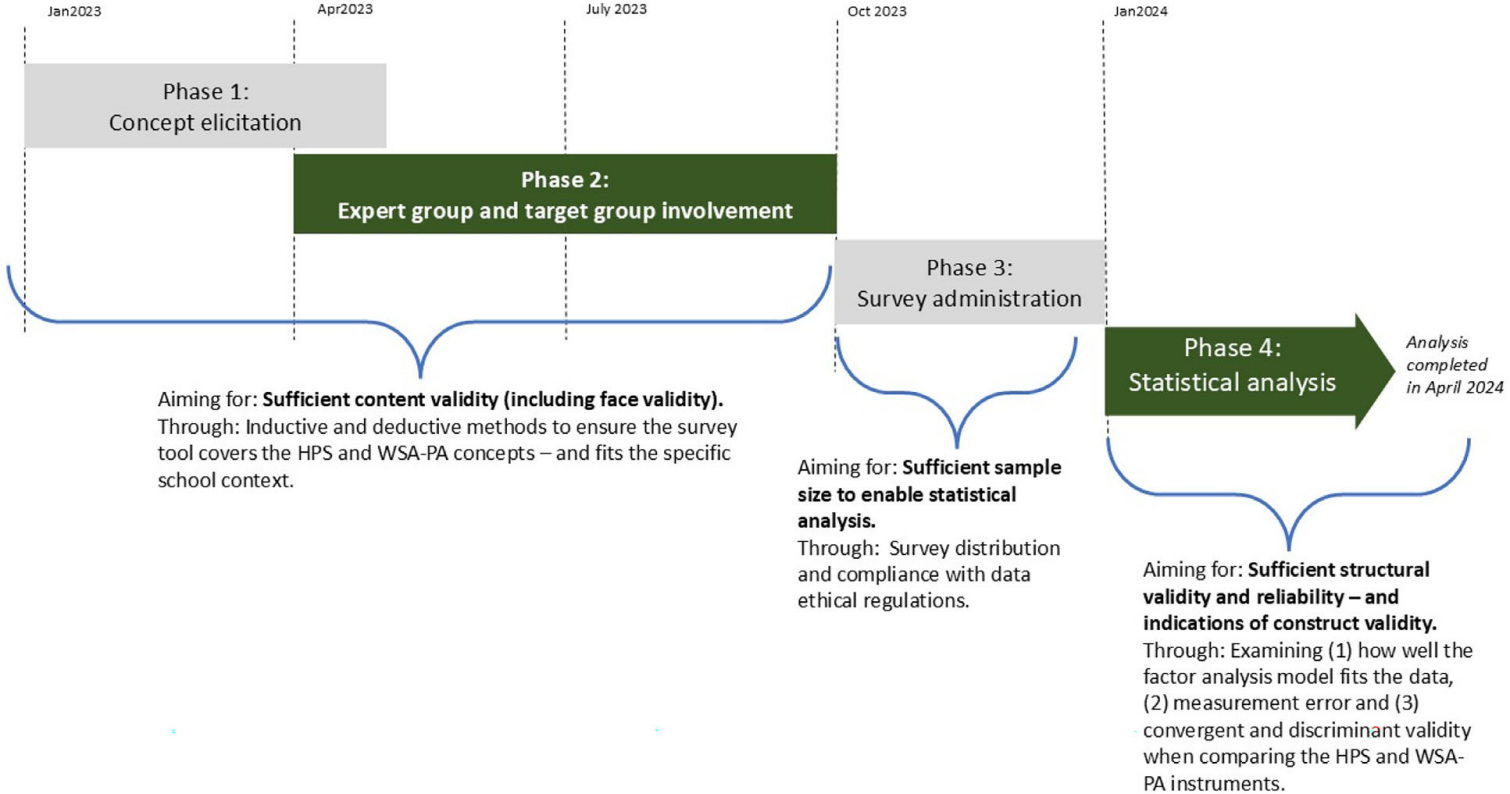
The four phases of this survey validation study. HPS, Health Promoting School; WSA‐PA, whole school approach to physical activity.

Inspired by the guidelines, the study was conducted in four phases: (1) concept elicitation, (2) involvement of expert and target groups, (3) survey administration, and (4) statistical (validation) analysis. An overview of these phases is presented in Figure 1. The subsequent Methods and Results sections are organized according to the phases.

#### 3.2.1 Phase 1: Concept Elicitation (January 2023 to April 2023)

The first phase aimed to elicit better and describe the concepts of HPS and WSA‐PA. As recommended [32], we worked with both deductive and inductive methods.

The deductive approach involved “desk research”. Specifically, we thoroughly examined three reviews within the relevant scope [1, 2, 4] and triangulated their various definitions and meanings. Through this triangulation, we developed a first draft HPS framework with core components to be measured by our survey tool. Since WSA‐PA relies on HPS, we used the same approach to elicit the WSA‐PA framework, supplementing it with components specifically related to physical activity, based on additional literature [10, 11, 33, 34, 35]. The next step in the deductive process involved analyzing existing school health‐related surveys and their items. We searched for Danish surveys reported in English that aligned with the proposed HPS and WSA‐PA frameworks. The rationale was to incorporate items that had been previously utilized whenever possible.

The inductive approach encompassed informal meetings/interviews with people who had experiences working with health promotion at specific Danish school settings (*n* = 3). For instance, we consulted with health department consultants from the Copenhagen Municipality who were supporting FGU schools in health‐promoting initiatives. These discussions aimed to gain insights into HPS and WSA‐PA practices and to understand the needs of the specific Danish schools.

The final proposed HPS and WSA‐PA frameworks comprised 10 overall headings and a pool of more than 150 potentially relevant survey items.

#### 3.2.2 Phase 2: Expert and Target Group Involvement (March 2023 to September 2023)

Content validity reflects the extent to which survey content adequately represents the construct it aims to measure [36]. Face validity can be considered a subdimension of content validity and concerns the comprehensibility and relevance of the items for the target group. As recommended [31, 32], we involved experts and the target group to ensure sufficient content and face validity.

The expert involvement comprised a Delphi‐inspired process with (1) Danish experts knowledgeable about health promotion and/or the specific FGU school context and (2) international experts in HPS and WSA‐PA. The Danish experts (*n* = 10) represented, for example, the Danish Ministry of Health and the FGU interest organization. The HPS experts (*n* = 6) represented the University College Southern Denmark, Maastricht University, Lyon University, and the Schools for Health in Europe (SHE) network foundation. All experts are recognized in the Acknowledgment section of this paper.

The Delphi process with national experts comprised three rounds.

During the first round (March 2023), we presented the tentative HPS and WSA‐PA framework at a meeting/group interview and the experts were asked to reflect upon the operationalization. For instance, the experts discussed whether the HPS framework made sense in the school context. The group interview (1 h 20 min) was recorded, and we used the recording and outputs from phase 1 to develop a first survey draft. During the second round (April–May 2023), the experts gave written feedback on the first survey draft. Then, we held four online group meetings, with 2–4 experts participating in each meeting, to discuss the survey comments. Based on comments and interviews, we drafted a second survey version. During the third round (September 2023), the experts reviewed the final survey version, provided written feedback, and approved the survey.

The Delphi‐inspired process with WSA‐PA international experts comprised one round (June–August 2023). The experts received a study description, including the second survey version in English. The experts provided written feedback on the survey; that is, on whether the operationalization sufficiently captured the HPS and WSA‐PA concepts and whether the specific items sufficiently covered the intended concepts. The assessors who understood Danish also appraised the Danish‐to‐English translations.

Based on the comments from HPS and WSA‐PA experts, a third survey draft was developed and pilot‐tested in two rounds. During the first round, *n* = 11 employees from four schools participated; in the second round, *n* = 8 employees from two schools participated. Practically, participants completed the questionnaire and afterward participated in a 30‐min online/telephone interview. After the first round, we made subsequent changes to the survey, while only minor changes were needed after the second round, and we concluded then that the survey had sufficient face validity. The piloting was completed in September 2023.

#### 3.2.3 Phase 3: Survey Administration (September 2023)

All Danish FGU institutions were invited to participate in the survey (*N* = 27). Initially, the school principal received an email invitation and a phone call. If the school principal agreed to participate, the school provided a list of email addresses for school staff, which were used to electronically distribute the questionnaire using SurveyXact. In the distribution email, participants received written information about the study's scope and the handling of personal data. Before starting the survey, participants consented by checking a box.

#### 3.2.4 Phase 4: Statistical Analyses

Most items were measured on 5‐point Likert scales, with rescaling applied to four items on the HPS scale and one item on the WSA‐PA scale (see Supporting Information: Appendices 2 and 3). Descriptive statistics were used to identify missing values, reverse‐coded items, and item variation.

Structural validity refers to the extent to which the scores obtained from an instrument accurately reflect the underlying dimensions of the construct being measured [36]. Structural validity was assessed using confirmatory factor analysis (CFA), which was chosen based on the hypothesized domains informed by earlier study phases. Robust estimations using the diagonally weighted least squares with mean and variance adjustment (WLSMV) method were applied to accommodate ordinal data and non‐normality [37]. Analyses were conducted with and without data imputation, but as results were consistent, only findings from non‐imputed data were reported. Additionally, second‐order CFA was performed to clarify the dimensionality of the constructs [32].

Model fit was evaluated using established indices, including the root mean square error of approximation (RMSEA), standardized root mean square residual (SRMR), comparative fit index (CFI), and Tucker–Lewis index (TLI) [32]. Thresholds for acceptable fit included RMSEA below 0.05, SRMR below 0.08, and CFI and TLI values above 0.95 (ibid). Further, a non‐significant chi‐square test was used to indicate overall model fit. Modification indices were reviewed to refine the model, allowing for covariances only when justified by theoretical considerations.

Following CFA, we examined factor loadings, variances, covariances, and residual error variances. Factor loadings above 0.5 were considered robust; variances were expected to be positive, with low residual error variances [38].

Reliability was assessed by examining internal consistency and inter‐rater reliability [36]. Internal consistency was measured using Cronbach's alpha (*α*) and omega hierarchical (*ω*_*h*_). The latter is recommended for multi‐dimensional factor structures and Likert‐scale data [39]. Internal consistency estimates above 0.7 were considered acceptable. Inter‐rater reliability was evaluated using the intraclass correlation coefficient (ICC) in a two‐way random effects model, with a threshold of ICC > 0.5 [31].

Construct validity, including convergent and discriminant validity, was evaluated using the multi‐trait multi‐method (MTMM) approach. This approach evaluates whether traits are positively correlated when measured by the same method (convergent validity) and whether unrelated traits show low correlations (discriminant validity). For this MTMM analysis, we defined traits as the subfactors of the HPS and WSA‐PA scales. Methods were specified as the measurement instruments (i.e., the entire HPS and WSA‐PA scales). We excluded subfactors with only one item (Subfactor 1 and Subfactor 7).

Specifically, we applied the Widaman (1985) approach to MTMM [40], which involves four steps [41]. In step 1, we specified a Correlated Traits/Correlated Methods model as the baseline for comparison with alternative CFA models. Step 2 involved comparing it with a Correlated Methods (no traits) model. A better fit for the baseline model supports convergent validity. In Step 3, we specified a perfectly correlated traits/freely correlated methods model, where traits are perfectly correlated (i.e., equal to 1), and method factors are freely estimated (in line with the baseline model). A significantly better fit for the baseline model supports discriminant validity. In Step 4, we compared a freely correlated traits/uncorrelated methods model with the baseline. A large difference in fit indicates poor discriminant validity across methods (ibid).

All statistical analyses were conducted in R using the Lavaan, Psych, and SemPlot packages.

### 3.3 Ethics

The study was approved by the Capital Region of Denmark (reference number: F‐23060092), which constitutes sufficient ethical clearance under Danish law. All data were processed and stored following the EU General Data Protection Regulation (GDPR) and national Danish data protection legislation.

## 4 Results

### 4.1 Operationalization—Outputs From Phase 1 and 2

Based on deductive and inductive processes, we developed scales to capture the HPS and WSA‐PA. Our operationalization of HPS identified seven subfactors: (1) school policies, (2) school ethos, (3) collaboration and involvement, (4) school practice, (5) quality of delivery, (6) physical and financial resources, and (7) school health services. The WSA‐PA scale comprises six subdimensions, excluding “school policies”. Table 1 outlines our operationalization, detailing specific items per subfactor, their derivation from literature or contextual adaptation, and the rationale behind their inclusion.

**TABLE 1:** Operationalization of the Health Promoting School (HPS) and the Whole School Physical Activity (WSA‐PA) into Survey Scales.

**Subfactor 1: school policies**	
Definition	A written policy, vision, or action plan regarding health and well‐being in general or in relation to specific health behaviors such as physical activity.
Evidence	Written policies are essential for anchoring health‐promoting initiatives within schools, as they provide a foundation for systematic implementation and consistency. Policies are a key indicator of the Health Promoting School (HPS) framework [1, 2, 3, 4, 5, 11, 33, 35]. In the Whole School Approach to Physical Activity (WSA‐PA), policies play a similarly critical role in fostering an environment that promotes physical activity among students [11, 35]. Evidence suggests that policies help institutionalize health‐related priorities and create sustainable practices aligned with broader educational goals [3].
Existing surveys	Existing HPS surveys operationalize school health policies in diverse ways. Some measure the number of policies and staff adherence [21, 27], while others focus on specific domains such as smoking restrictions, fit‐aid kits, and medication administration records [23]. Broader aspects, such as job satisfaction, health facilities, and extracurricular activities, have also been included in policy measures [26]. Operationalizations may also emphasize emergency preparedness or systems for emergency cases [23, 26]. For physical activity policies, researchers have developed structured tools addressing areas such as physical education, recess, and extracurricular programs [42]. Other approaches assess teacher awareness and involvement in policy implementation, focusing on knowledge, communication, and collaboration [43]. Assessments may also include requirements for physical education minutes, facilities, and integration of physical activity into daily school routines [29].
Rationale	Our operationalization assesses whether schools have written policies, visions, or action plans addressing specific health‐related topics, including physical activity, without focusing on the specific details of these policies. By emphasizing general policy presence rather than specific content, we aim to reflect the role of policies in establishing a foundation for health promotion.

**Operationalization of subfactor 1**		
**Item no.**	**HPS scale**	**WSA‐PA scale**
1	Aggregate item that assesses the number of written school policies, visions, or action plans concerning health and well‐being at the school (see Supporting Information: Appendix 2 for specific items).	Not included in the final WSA‐PA scale.

**Subfactor 2: school ethos**	
Definition	The school ethos reflects fundamental values concerning health and well‐being in general or in relation to specific health behaviors such as physical activity. This encompasses shared beliefs, practices, and management approaches.
Evidence	The ethos of a school plays a pivotal role concerning the HPS, serving as the foundation for a supportive environment. Key indicators in the HPS framework include recognizing health promotion as a school responsibility and understanding the reciprocal relationship between health and education [1, 4, 5]. A positive and inclusive psychosocial climate, free from bullying and discrimination is also highlighted as essential [1, 4]. Moreover, management and leadership practices that foster shared values and joint responsibilities are crucial for achieving a supportive school ethos [1, 2, 3, 4].
Existing surveys	Existing surveys capture school ethos through various dimensions. Some focus on leadership and management roles, including change management and shared health‐related values [20]. Others assess broader psychosocial and pedagogical factors [21, 26, 27].
Rationale	Our operationalization aims to align with the evidence and approaches found in prior surveys and covers: Core values that explicitly promote health and well‐being or physical activity. Leadership's role in setting goals and direction for health and well‐being or physical activity. A shared understanding among staff of the interconnection between health and education. The presence of an inclusive psychosocial climate defined as zero tolerance for bullying or discrimination.

**Operationalization of subfactor 2**		
**Item no.**	**HPS scale**	**WSA‐PA scale**
2	Promoting students' health and well‐being is part of the school's core values.	Promoting students' physical activity is part of the school's core values.
3	The school is responsible for promoting health and well‐being among students.	The school is responsible for promoting physical activity among students.
4	The school has zero tolerance for bullying/online bullying and discrimination.	Not included in the final WSA‐PA scale.
5	The school staff's practice is based on the understanding that there is a link between students' learning and their health and well‐being.	The school staff's practice is based on the understanding that there is a link between students' learning and their physical activity.
6	The management sets goals and direction for the school's work with health and well‐being.	The management sets goals and direction for the school's work with physical activity.

**Subfactor 3: Collaboration and involvement**	
Definition	Collaboration and involvement reflect the engagement of students, staff, and external stakeholders concerning health and well‐being in general or in relation to specific health behaviors such as physical activity.
Evidence	The involvement of the entire school community—staff and students—is a cornerstone of the Health Promoting School (HPS) framework [1, 2, 3, 4, 5, 7]. This approach emphasizes that active engagement and collaboration among actors within the school enhance the school's capacity to implement sustainable health promotion strategies effectively. The Schools for Health in Europe (SHE) network further emphasize the importance of creating a pedagogical environment grounded in the active participation of students [4]. This approach ensures that students are not merely recipients but also contributors to the health‐promoting processes, fostering ownership and sustainability in HPS implementation. Collaboration with local community stakeholders, such as local governments, non‐governmental organizations, and businesses, is also a key HPS indicator [1, 2, 3, 4, 5, 7]. The rationale is that schools should capitalize on the resources in their neighborhood to create better conditions for health and well‐being. Namely, active engagement, consultation, and partnerships with local actors foster better HPS implementation. HPS frameworks also highlight the importance of including parents as stakeholder [1, 3, 4].
Existing surveys	Involvement of the school community is consistently measured in HPS surveys [10, 20, 22, 23, 24, 26, 27], with variations in specific approaches. Explicit measurement of students' active participation appears to be less common.
Rationale	In the context of Danish FGU schools, where most students are over 18 years old, parental involvement was deemed less relevant and therefore excluded from our operationalization. This decision aligns with the specific context, ensuring that the operationalization remains focused on stakeholders who have a direct and active role in the school environment. Our operationalization is structured to capture: Staff involvement in shaping school practices related to health and well‐being or physical activity. Student involvement in shaping school practices related to health and well‐being or physical activity. Integration of active participation principles in teaching and school activities. External collaborations at both individual and structural levels. Through pilot testing, we identified the need to distinguish between two forms of external collaboration: (1) Individual‐oriented initiatives: These are targeted programs addressing personal needs or challenges, such as counseling: (2) Structural initiatives: These are programs targeting groups at students or more structural initiatives indirectly influencing students such as policy advocacy, community events, or competence development. This distinction allows us to capture the varying practices across schools and to provide a nuanced understanding of collaboration and involvement.

**Operationalization of subfactor 3**		
Item no.	HPS scale	WSA‐PA scale
7	Staff are involved in deciding or developing the school's practice regarding health and well‐being.	Staff are involved in deciding or developing the school's practice regarding physical activity.
8	Students are involved in deciding or developing the school's practice regarding health and well‐being.	Students are involved in deciding or developing the school's practice regarding physical activity.
9	The school collaborates with external stakeholders on individual‐targeted initiatives regarding health and well‐being.	The school collaborates with external stakeholders on individual‐targeted initiatives regarding physical activity.
10	The school collaborates with external stakeholders on other than individual‐targeted initiatives regarding health and well‐being.	The school collaborates with external stakeholders on other than individual‐targeted initiatives regarding physical activity.
11	School practices, including teaching and other activities, are based on active participation and involvement of students.	Not included in the final WSA‐PA scale.

**Subfactor 4: school practice**	
Definition	School practice involves the integration of health and well‐being practices or physical activity into formal or informal school activities. This includes embedding health‐related practices into planned educational practices or spontaneous interactions during the school day.
Evidence	The integration of health and well‐being into the school's everyday practices is a key indicator of the Health Promoting School (HPS) framework [1, 2, 3, 4, 5, 7]. The HPS approach adopts a school‐based “add‐in” philosophy, meaning that health and well‐being are seamlessly incorporated into existing educational activities rather than treated as additional or separate components [21, 44].
Existing surveys	To our knowledge, no HPS surveys have specifically measured the extent to which health and well‐being are integrated into school practices, although some surveys indirectly cover aspects of the integration. Regarding physical activity, some questionnaires provide detailed mappings, such as the content of physical activity lessons [28]. Others more broadly assess the integration of physical activity into lessons and the frequency of for example, energizers [45].
Rationale	We operationalized this as the extent to which staff incorporates health‐related topics into practices during the school day. Pilot testing revealed the importance of distinguishing between: Organized practices: These include structured activities, such as integrating health‐related topics into lessons or other planned activities. Informal practices: These encompass unplanned interactions, such as casual conversations with students about health and well‐being in general or physical activity specifically.

**Operationalization of subfactor 4**		
**Item no.**	**HPS scale**	**WSA‐PA scale**
12	An aggregate item assessing how often staff integrate various health and well‐being areas into organized activities during school time (see Supporting Information: Appendix 2 for specific items).	How often do you work with organized or planned activities related to physical activity during the school day?
13	An aggregate item assessing how often staff integrate various health and well‐being areas into informal activities during school time (see Supporting Information: Appendix 2 for specific items).	How often do you have informal conversations with students about physical activity during the school day?

**Subfactor 5: quality of delivery**	
Definition	Quality of delivery involves the quality of the school's practice concerning health and well‐being in general or concerning specific health behaviors such as physical activity.
Evidence	The need for staff to possess the right skills, competencies, and motivation is emphasized in HPS documents [2, 3, 4]. The rationale is that well‐equipped and motivated staff are essential for delivering high‐quality practices concerning health and well‐being. Using data and evidence to develop and monitor health‐related school practices is another key HPS indicator [1, 4]. Building practices on local needs (e.g., data) and evidence ensures a more relevant and potentially effective approach. Linking schools with local communities through health‐promoting initiatives further supports these goals [1, 4]. Regarding physical activity, such connections can encourage students to participate in organized sports and adopt active transportation to and from school [4, 11, 35]. Additionally, focusing on enhancing students' action competencies and health literacy is also highlighted as a crucial aspect of HPS [4].
Existing surveys	Staff competencies or motivation have been measured in some existing surveys with varying approaches [20, 21, 27]. Some HPS surveys also address data‐related aspects [21, 27], while only a few instruments capture health literacy approaches [27]. Regarding physical activity, existing surveys measure links between schools and communities in promoting physical activity, such as access to community physical activity programmes and active transportation [46].
Rationale	We operationalized quality of delivery to capture: Staff readiness: This involves assessing the skills, knowledge, and motivation of staff. Evidence‐based and tailored practices: This captures the extent to which practices are informed by evidence and local needs. Student empowerment: This measures efforts to enhance students' action competencies and health literacy. Community links: This assesses how schools encourage connections with for example, extracurricular activities, and local sports clubs or promote active transportation.

**Operationalization of sub‐factor 5**		
**Item no.**	**HPS scale**	**WSA‐PA scale**
14	I have sufficient skills and knowledge to work with health and well‐being.	I have sufficient knowledge and skills to work with physical activity.
15	An aggregate item that assesses the extent to which staff are motivated to work with health and well‐being (see Supporting Information: Appendix 2 for specific items).	To what extent are you motivated to work with physical activity?
16	To what extent is your practice regarding health and well‐being based on the best available knowledge?	To what extent is your practice concerning physical activity based on the best available knowledge?
17	To what extent is your practice regarding health and well‐being based on local knowledge and needs?	To what extent is your practice concerning physical activity based on local knowledge and needs?
18	In your daily work, to what extent do you seek to increase students' action competence and health literacy, that is, their ability to access, understand, and act on information related to health and well‐being?	Not included in the final WSA‐PA scale.
19	To what extent do you encourage students to participate in leisure time activities?	To what extent do you encourage students to be physically active during leisure time?
19b	Not included in the final HPS scale.	To what extent do you encourage students to use active transportation to and from school (e.g., bicycling)?

**Subfactor 6: physical and financial resources**	
Definition	Physical and financial resources reflect the adequacy of the school's facilities and funding to support health and well‐being practices or specific practices such as physical activity. This includes the availability of suitable environments, resource allocation, and investments in staff development.
Evidence	Sufficient facilities, such as outdoor areas and premises for health‐promoting initiatives, are recognized as key HPS indicators [1, 4, 5, 11, 35]. Resource allocation is also emphasized as a prerequisite for implementing HPS practices [1, 2, 3, 4]. This includes providing time for staff to engage in health‐promoting activities, funding new initiatives, and opportunities for staff competency development. The rationale is that appropriate facilities and allocated resources enable schools to support the initiation and sustainability of health‐promoting practices.
Existing surveys	Many HPS surveys measure physical facilities to varying extents [20, 23, 24, 26, 27]. These surveys often focus on specific elements such as water, toilets, and safety [23, 24, 26] or facilities related to nutrition and physical activity [20, 26, 27]. Few surveys directly assess financial resource allocation aspects.
Rationale	Our operationalization aims to provide a broad assessment of physical and financial resources by focusing on: Physical environment: Evaluating whether the overall physical environment is suitable for health and well‐being or physical activity. Resource availability: Assessing financial and time resources allocated to health‐promoting or physical activity. Staff development: Measuring management's prioritization of competency development regarding health and well‐being or physical activity.

**Operationalization of subfactor 6**		
**Item no.**	**HPS scale**	**WSA‐PA scale**
20	The school's physical environment is suitable for working with health and well‐being.	The school's physical environment is suitable for working with physical activity.
21	The school has the financial resources needed for working with students' health and well‐being.	The school has the financial resources needed for working with students' physical activity.
22	The management prioritizes competence development regarding health and well‐being.	The management prioritizes competence development concerning physical activity.
23	I have sufficient time to work on students' health and well‐being.	I have sufficient time to work with students' physical activity.

**Subfactor 7: school health services**	
Definition	School health services reflect the facilitation of services concerning health and well‐being in general or specifically in relation to physical activity. This includes the availability of personnel, programs, and support systems for addressing students' health‐related needs.
Evidence	Facilitating school health services, either on the school campus or within the school's neighborhood, is a key HPS indicator [1, 4, 5, 6]. These services are critical in ensuring that students have access to necessary health‐related support.
Existing surveys	Many HPS surveys address school health services, though items vary considerably. Some surveys assess the presence of health cards, first‐aid kits, and first‐aid training [24]. Others evaluate screening systems, the presence of healthcare professionals, and health‐related teacher training [22, 23]. Additionally, some instruments measure student health monitoring practices, the presence of school nurses, and vaccination checks [26]. To our knowledge, no previous surveys have explicitly measured health services related to physical activity.
Rationale	Our operationalization focuses on respondents' awareness of school health services – generally and specifically related to physical activity. Instead of detailing specific services, this approach measures the extent to which schools enable health‐related support for students. The rationale is to assess the actual implementation of whether students who require assistance can easily be connected to appropriate resources through the school.

**Operationalization of subfactor 7**		
Item no.	HPS scale	WSA‐PA scale
24	Do you know whom you can refer students to if they experience health or well‐being‐related issues? for example, someone connected to the school or an external professional.	Do you know who you can refer students to if they want to be more physically active? for example, someone connected to the school or an external professional.

The operationalization framework is further supported by statistical analyses detailed under *Structural validity, construct validity*, *and reliability results—Outputs from Phase 4*. After validation (Phase 4), the HPS scale comprises 24 items, while the WSA‐PA scale comprises 21 items. A detailed usage guide for these survey tools can be found in Supporting Information: Appendices 2 and 3, which also include the original Danish survey items.

### 4.2 Sample Size and Participants—Outputs From Phase 3

A total of 25 FGU institutions participated in the survey (institution participation rate: 91.8%), comprising 78 physical school addresses. Altogether, *n* = 921 staff participated in the survey (response rate: 72%).

Due to missing values, we conducted the factor analysis on *n* = 453 observations for the HPS and *n* = 418 for the WSA‐PA. The recommended sample size for CFA is at least 300 or 5 times the number of items included in a survey construct [31, 32]. Hence, the sample size was sufficient for CFA.

Participant characteristics are included in Table 2. On average, participants were 48.9 years old, and 41% were male. The included sample differed significantly from the full sample in certain background characteristics (see Table 2).

**TABLE 2:** Participant characteristics.

Characteristic	All participants	Included HPS	Included WSA‐PA
*N* individuals	921	453	418
Age, mean (SD)	48.9 (10.1)	49.1 (10.1)	48.7 (10.1)
Male gender, %	41.0%	43.3%	45.9%*
Educational level, %			
Primary, secondary, short higher, or other	28.2%	26.9%	25.8%
Medium higher	54.9%	57.0%	56.5%
Long higher	16.8%	16.1%	17.7%
Years employed at school^a^, median (IQR)	4 (2;4)	**4** ^**b**^ (2;4)**	4 (2;4)
Function			
Teacher, general subjects	31.9%	32.7%	35.4%
Teacher, vocational subjects	45.2%	45.3%	44.0%
Counselor, resource person, or other function	23.0%	22.2%	20.7%
Special role in relation to health and wellbeing	14.0%	18.3%**	18.4%**

*Note*: Bold font indicates a significant difference between the included and the non‐included populations; *p < 0.05, **p < 0.001.

^a^ Because FGU was established in 2019, the maximum employment length at the time of data collection was 4 years.

^b^ Although medians (IQR) were identical, statistical testing showed that included participants had been employed significantly longer compared to all participants.

### 4.3 Structural Validity, Construct Validity, and Reliability Results—Outputs From Phase 4

Results from the CFA, that is, the fit indices, are shown in Table 3. These estimations are based on model adjustments. Specifically, on both the HPS and WSA‐PA scales, covariances were added between the following items: In the HPS scale, covariances were added between items 7–8, related to the involvement of students and staff, and between items 9–10, concerning collaboration with external stakeholders. Similarly, on the WSA‐PA scale, covariances were added between items 19 and 19b, which both pertain to encouraging physical activity in leisure time. Importantly, these added covariances align with theoretical expectations.

**TABLE 3:** Structural validity results, that is, goodness of fit based on Confirmatory Factor Analysis (CFA).

	Estimates first order CFA		Estimates second order CFA	
	HPS scale	WSA‐PA scale	HPS scale	WSA‐PA scale
Root mean error of approximation	0.023	0.043	0.037	0.062
Standardized root mean square residual	0.051	0.062	0.058	0.076
Comparative fit index	0.992	0.981	0.979	0.950
Tucker–Lewis index	0.991	0.976	0.977	0.957
Chi‐Square (df)	7506 (276)	7002 (210)	7506 (276)	7002 (210)
*p*‐value	0.007	0.001	0.001	0.001

Abbreviations: HPS, Health Promotion School; WSA‐PA, Whole School Approach Physical Activity.

As detailed in Table 3, the fit indices for the HPS scale indicate good to excellent results, particularly the estimates from the first‐order factor analysis (e.g., RMSEA = 0.023 and CLI = 0.992). Similarly, the fit estimates for the WSA‐PA scale are good, with the best results from the first‐order factor analysis (e.g., RMSEA = 0.043 and CLI = 0.981). Results from the second‐order factor analysis are acceptable (e.g., RMSEA = 0.037 and CLI = 0.979 for HPS; and RMSEA = 0.062 and CLI = 0.950 for WSA‐PA). It is noted that the Chi‐square test did not meet the criterion for non‐significance. However, the Chi‐square test is sensitive to sample size, and with larger samples, it tends to become significant even when the model fits the data well [47]. Therefore, despite the Chi‐square being significant, the other fit indices suggest that both scales meet the threshold criteria for structural validity.

The factor structure diagrams detailing factor loadings and residual error variances are available in Supporting Information: Appendix 4. It was confirmed that all variances and covariances were positive, indicating meaningful relationships between the latent variables within both the HPS and WSA‐PA scales. Most factor loadings exceeded the recommended threshold of 0.5. Specifically, factor loadings for the HPS ranged from 0.40 to 0.83, while those for the WSA‐PA ranged from 0.46 to 0.83. This robustness in factor loadings and the positive variances and covariances further support the structural validity (and reliability) of the HPS and WSA‐PA scales.

The reliability results are summarized in Table 4. Internal consistency assessed using Cronbach's Alpha (*α*) and Omega Hierarchical (ωh) yielded acceptable values ranging between 0.56 and 0.9 across the HPS and WSA‐PA scales. Inter‐rater reliability measured using the Intraclass Correlation Coefficient (ICC) yielded values above 0.5 for all subfactors, which is satisfactory. In summary, the reliability estimates for the overall HPS and WSA‐PA scales were acceptable.

**TABLE 4:** Reliability results.

	Cronbach's alpha (*α*)		Omega hierarchical (ωh)		Intraclass correlation coefficient	
	HPS scale	WSA‐PA scale	HPS scale	WSA‐PA scale	HPS scale	WSA‐PA scale
Subfactor 1: School policies	NA^a^	NA^a^	NA^a^	NA^a^	NA^a^	NA^a^
Subfactor 2: School ethos	0.82	0.82	0.78	0.82	0.791	0.809
Subfactor 3: Collaboration and involvement	0.78	0.82	0.68	0.66	0.777	0.801
Subfactor 4: School practice	NA^a^	NA^a^	NA^a^	NA^a^	0.511	0.676
Subfactor 5: Quality of delivery	0.72	0.75	0.58	0.56	0.688	0.732
Subfactor 6: Physical and financial resources	0.72	0.76	CNBD^b^	0.75	0.711	0.773
Subfactor 7: School health services	NA^a^	NA^a^	NA^a^	NA^a^	NA^a^	NA^a^
Total	0.9	0.89	0.68	0.65	0.85	0.867

Abbreviations: HPS, Health Promotion School; WSA‐PA, Whole School Approach Physical Activity.

^a^ Not applicable, given the few items in the subscale. Also, note that subfactor 1 is not part of the WSA‐PA scale.

^b^ Could not be determined due to overparameterization, as indicated by negative degrees of freedom (−3) and a fix index of 0.

The model comparisons based on the MTMM analysis are available in Supporting Information: Appendix 5. In summary, the correlated traits/correlated methods model (Model 1) significantly outperforms both the Correlated Methods (no traits) model (Model 2) and the perfectly correlated traits/freely correlated methods model (Model 3), demonstrating evidence of convergent and discriminant validity. This suggests that the subfactors of the HPS and WSA‐PA scales effectively measure distinct and important aspects of the overall latent variable. Additionally, the Correlated Traits/Correlated Methods model (Model 1) is significantly better than the Freely Correlated Traits/Uncorrelated Methods model (Model 4). Ideally, there should be no difference to fully support discriminant validity across measurement methods. We also note that the models based on the MTMM do not meet the threshold criteria for most goodness of fit indices. Although better model fits are preferred, model comparisons are the most relevant analysis to evaluate construct validity in the Widaman approach to MTMM.

## 5 Discussion

This study aimed to develop and validate two survey instruments: one designed to assess the core indicators of the HPS and another tailored to capture the Whole School Approach to Physical Activity (WSA‐PA). Additionally, the study sought to produce guidelines—and detail our operationalization—to facilitate using these instruments in research and practice.

### 5.1 Summary of Main Findings

We successfully developed and validated instruments for assessing HPS and WSA‐PA practices and capacity. The HPS scale consists of seven subdimensions: (1) school policies, (2) school ethos, (3) collaboration and involvement, (4) school practice, (5) quality of delivery, (6) physical and financial resources, and (7) school health services. The WSA‐PA scale comprises six subdimensions, excluding “school policies.” Both instruments demonstrated strong psychometric properties, with good‐to‐excellent fit indices and robust factor loadings supporting their structural validity. Reliability analyses showed acceptable internal consistency and inter‐rater reliability, further confirming their overall robustness and utility.

The MTMM analysis provided indicative evidence of construct validity, that is, convergent and discriminant validity. Hence, the HPS and WSA‐PA scales measure distinct and significant traits (subfactors). However, the MTMM analysis also indicates that WSA‐PA and HPS are closely related; challenging their discriminant validity.

### 5.2 Reflections on Methods and Operationalization

To develop our questionnaires, we synthesized materials from recent reviews, expert insights, and feedback from the target population to identify core HPS and WSA‐PA indicators. This process was designed to address the longstanding variability in how the HPS framework has been operationalized across different contexts. Briefly, the WHO introduced a framework of the concept in 1996, containing six indicators [5]. Over time, researchers expanded and refined this framework: Langford et al. highlighted three broad pillars of HPS for a Cochrane review in 2014 [7], while Samdal and Rowling identified key implementation factors in their comprehensive reviews from 2011 to 2015 [2, 3]. Most recently, in 2021, both the WHO and the Schools for Health in Europe (SHE) network updated their respective frameworks to include distinct sets of eight HPS indicators [1, 4].

Although the underlying principles of HPS remain consistent, these varying operationalizations present challenges for standardization and cross‐contextual application [30]. Our approach aimed to overcome these challenges by integrating elements from multiple frameworks into a cohesive and practical tool. By triangulating theoretical advancements and adapting them to school‐level needs, we developed instruments that are both robust and adaptable. This methodology aligns with those of Lee (2014) [23], who similarly drew on earlier WHO conceptualizations to create a HPS questionnaire.

In this study, we focused on measuring HPS and WSA‐PA practices and capacity, building on the WHO classical definition of a HPS as one that “constantly strengthens its capacity as a healthy setting for living, learning, and working” [1]. Practices refer to the actions undertaken by schools, while capacity reflects their ability to sustain and develop these actions. These concepts often overlap; for instance, engaging staff and students in health‐related activities is both a practice and evidence of capacity. Our operationalization further drew on prior research, incorporating elements such as adherence, magnitude (dose), and quality of delivery, inspired by a recent HPS implementation survey [21]. By emphasizing intuitive and contextually relevant subfactors, we ensured our instruments capture not only what schools do but also how effectively they do it.

Many existing HPS questionnaires lack clarity in their focus, conflating general indicators, specific health behaviors, and facilities. Although some recent tools aim to measure “implementation” [21] or “culture” [20], these terms might be abstract and difficult to comprehend for schools. We chose to focus on practices and capacity as they offer clearer and more actionable insights.

Developing questionnaires for the HPS and WSA‐PA presented challenges due to the comprehensiveness and complexity of these concepts, leading to both theoretical and practical considerations.

Theoretically, both the HPS framework and school contexts reflect complex systems involving multiple interacting levels—individual, professional, procedural, and policy—that adapt and co‐evolve over time [1, 48, 49, 50]. Thus, any attempt to quantify the HPS framework inherently simplifies its dynamic nature. Our approach aimed to balance this complexity with the need for measurable and monitorable constructs.

Practically, the length of the questionnaire was a critical concern. Comprehensive frameworks like HPS and WSA‐PA often require extensive items to capture all dimensions, but brevity is essential to accommodate busy school schedules. To address this, we prioritized core subdimensions and excluded aspects like school personnel's well‐being, which, while important, fell outside our immediate focus. These decisions were validated through literature review and expert feedback. Existing HPS surveys range from 18 to 138 items [10, 20, 21, 22, 23, 24, 25, 26, 27] (averaging 42), whereas the WHO and CDC instruments exceed 100 items, limiting their practicality. Our HPS survey includes 24 items, and the WSA‐PA survey contains 21 items, achieving a balance between depth and usability.

By addressing these challenges, we developed tools that provide a practical yet comprehensive way to assess HPS and WSA‐PA practices and capacity, making them accessible for diverse educational settings while maintaining their theoretical integrity.

### 5.3 Implications for School Health Policy, Practice, and Equity

To ensure practical applicability, we designed the questionnaires with a focus on clarity and usability. Detailed guidance for researchers and practitioners is provided in Supporting Information: Appendices 2 and 3, making the tools more accessible for real‐world use. The relatively short length of the questionnaires enhances their feasibility for schools, policymakers, and researchers alike.

The tools are designed to support schools in refining their health‐promoting practices, inform policymaking, and contribute to research on HPS and WSA‐PA implementation. By identifying areas for improvement, the questionnaires can help promote health equity through better implementation of HPS and WSA‐PA. Specifically, the WSA‐PA scale is already being used in an upcoming Danish systems‐based intervention study to enhance physical activity at FGU schools.

Our factor analyses underscore the validity of sub‐factor scores, providing more actionable insights than a single aggregate score. Sub‐factors allow for targeted interventions. Aggregate HPS and WSA‐PA scores can also be used, as supported by our findings.

Although developed in Denmark for FGU schools, we believe these tools have broader applicability. Tailoring the questionnaires to local needs, such as including questions on parental involvement or specific health services, may enhance their relevance in different educational settings. Variations in legislation and cultural practices should also be considered when adapting the tools to new contexts.

Lastly, while the WSA‐PA survey is particularly relevant for settings where physical activity is a strategic focus, future adaptations could emphasize other health behaviors, such as nutrition or well‐being. The flexibility and transparency of these developed tools allow for customization to meet the priorities of specific health‐promoting initiatives. However, further validation would be needed for such tailoring.

#### 5.3.1 Strengths and Limitations

A key strength of this study is its adherence to established guidelines for developing and validating survey instruments. This rigorous approach included triangulating knowledge from literature, incorporating expert input, and engaging the target population. These steps ensured the questionnaires are both theoretically robust and contextually relevant. Statistical methods were employed to evaluate dimensionality, reliability, and construct validity, ensuring comprehensive and transparent validation. Compared to some prior HPS validations, the comprehensive process here represents a significant balance between conceptual rigor and methodological strength.

However, some limitations must be acknowledged. A key limitation is that certain sub‐factors within the HPS and WSA‐PA scales contain only one or two items. While these “solo” sub‐factors are theoretically justified, future surveys may benefit from expanding these dimensions to include additional relevant items, such as more detailed questions about school health services and policies.

Additionally, our operationalization of school policies was limited. We measured the number of existing policies but did not assess how practices align with written policies. This limitation also meant that we could not include the sub‐factor “school policies” in the WSA‐PA scale. A more meaningful approach might involve evaluating the extent to which practices are guided by formal policies. This is an area for improvement in future iterations.

The limitations also extended to using the MTMM approach, as the “solo” sub‐factors were excluded from the analysis. This omission likely contributed to the less satisfactory goodness‐of‐fit indices observed. The MTMM results should be considered indicative rather than definitive evidence of discriminant and convergent validity.

Finally, the survey was tested using a cross‐sectional design in a single population (Danish FGU schools). To strengthen test–retest reliability and applicability, replication across diverse contexts and multiple time points is necessary. Future studies should explore these aspects to enhance the robustness and generalizability of the instruments.

## 6 Conclusion

We have developed instruments to assess HPS and WSA‐PA practices and capacity, which demonstrate strong validity and acceptable reliability. Additionally, we have provided a comprehensive operationalization that clarifies our decisions and exclusions, along with guidelines to facilitate the usability of these instruments in research and practice.

This work is not the definitive solution for measuring HPS or WSA‐PA. The inherent complexity of these constructs poses ongoing measurement challenges. Nonetheless, this study offers interpretable and theoretically consistent questionnaires that can serve as a basis for further research and development.

## Conflicts of Interest

The authors declare no conflicts of interest.

## Supporting information


**Data S1:**josh70071‐sup‐0001‐Supinfo.docx.

## Data Availability

The data that support the findings of this study are available on request from the corresponding author. The data are not publicly available due to privacy or ethical restrictions.
