# Supplementary material for: A Novel Survey for Assessing Health‐Promoting School Practices and Capacity: Validation and Adaptability to Physical Activity
Source: J Sch Health. 2025 Sep 4;95(12):1047–59. doi: 10.1111/josh.70071 (PMC12621173; doi:10.1111/josh.70071)
Supplement: Supplementary file 1 — Data S1:josh70071‐sup‐0001‐Supinfo.docx. [file JOSH-95-1047-s001.docx]

**Supplementary Files 1- 5**

**Appendix 1**

| **Concept** | **Guideline by Boateng et al. 2018** | **Guideline by Mokkink et al. 2017** | **How this scale development and validation study adhered to the guidelines** |
| --- | --- | --- | --- |
| **Design and domain specification** | | |  |
| General design requirements (Mokkink et al. 2017); Domain specification (Boateng et al. 2018) | 1.1 Specify the purpose of the domain | 1.1 Is a clear description provided of the construct to be measured? | We clarify the rationale of the study in *Introduction* and clearly describe the aim of the study. In the *Setting* section we describe the target population and the context of FGU schools. We further discuss the broader applicability of the survey in the Discussion. |
|  | 1.2. Confirm that there are no existing instruments | NA |  |
|  | NA | 1.2. Is the origin of the construct clear: was a theory, conceptual framework or disease model used or clear rationale provided to define the construct to be measured? |  |
|  | NA | 1.3. Is a clear description provided of the target population for which the [survey] was developed? |  |
|  | NA | 1.4. Is a clear description provided of the context of use |  |
| **Concept elicitation and item generation** | | |  |
| Item generation (Boateng et al. 2018);  Concept elicitation (Mokkink et al. 2017) | 1.3. Describe the domain and provide preliminary conceptual definition 1.4. Specify the dimensions of the domain if they exist a priori. 1. 5. Define each dimension | NA | We used deductive methods, including a literature review and an examination of existing surveys, as well as inductive methods, such as informal meetings and interviews with stakeholders. Through this process, we developed a conceptual working definition of HPS and WSA-PA and identified a pool of potentially relevant items. These aspects are described in detail under Methods – Phase 1: Concept Elicitation.  We did not employ rigorous qualitative methods to systematically derive themes for the survey, as this was not considered necessary for this study. |
|  | 1.6 Deductive methods: literature review and assessment of existing scales | NA |  |
|  | 1.7 Inductive methods: exploratory research methodologies including focus group discussions and interviews | 6. Was an appropriate qualitative data collection method used to identify relevant items for a new [survey]? 7. Were skilled group moderators/interviewers used? 8. Were the group meetings or interviews based on an appropriate topic or interview guide? 9. Were the group meetings or interviews recorded and transcribed verbatim? 10. Was an appropriate approach used to analyse the data? 11. Was at least part of the data coded independently? 12. Was data collection continued until saturation was reached? 13. For quantitative studies (surveys): was the sample size appropriate? |  |
|  | NA | *Note: Section 1 (14-35) "Cognitive interview study" is not included in this table.* | We did not use cognitive interviews as part of the inductive concept elicitation phase. |
| **Content validity** | | |  |
| Asking patients and professionals about relevance (Mokkink et al. 2017);  Evaluation by experts and target group (Boateng et al. 2018) | 2.1 Quantify assessments of 5-7 expert judges using formalized scaling and statistical procedures including content validity ratio, content validity index, or Cohen’s coefficient alpha | NA | In this study, we refined our preliminary operationalization through a Delphi-inspired process involving two types of experts: Danish experts with in-depth knowledge of practice and health promotion at FGU schools, and international experts specializing in HPS and WSA-PA. The process included three rounds with the Danish experts and one round with the international experts. The number of experts (n=16) is considered acceptable. The Delphi-inspired process is detailed under *Methods, Phase 2 Expert and target group involvement.* Rather than following a full Delphi consensus-seeking procedure (including statistical procedures), our approach was Delphi-inspired, incorporating feedback, input, and expert interviews to strengthen the survey operationalization. |
|  | 2.2 Conduct Delphi method with expert judges | 2.22 + 2.27: Was an appropriate method used to ask professionals whether each item is relevant for the construct of interest? Was an appropriate method used for assessing the comprehensiveness of the [survey]? 2.23 + 2.28: Were professionals from all relevant disciplines included? 2.24 + 2.29: Was each item tested in an appropriate number of professionals? (N> 7 good, N= 4-6 adequate) 2.25 + 2.30: Was an appropriate approach used to analyse the data? 2.26 + 2.31: Were at least two researchers involved in the analysis? |  |
|  | 2.3 Conduct cognitive interviews with end users of scale items to evaluate face validity  3.1 Administer draft questions to 5–15 interviewees in 2–3 rounds while allowing respondents to verbalize the mental process entailed in providing answers | 2.1 + 2.8 + 2.15: Was an appropriate method used to ask patients whether each item is relevant for their experience with the condition? Was an appropriate method used for assessing the comprehensiveness of the [survey]? Was an appropriate qualitative method used for assessing the comprehensibility of the [survey instructions, items, response options, and recall period? 2.2 + 2.9 + 2.16: Was each item tested in an appropriate number of patients? (N> 7 good, N= 4-6 adequate) 2.3 + 2.10 + 2.17: Were skilled group moderators/interviewers used? 2.4 + 2.11 + 2.18: Were the group meetings or interviews based on an appropriate topic or interview guide? 2.5 + 2.12 + 2.19: Were the group meetings or interviews recorded and transcribed verbatim? 2.6 + 2.13 + 2.20: Was an appropriate approach used to analyse the data? 2.7 + 2.14 + 2.21: Were at least two researchers involved in the analysis? | We conducted two rounds of pilot testing among the target group, involving a total of 19 staff members, which is deemed appropriate. Rather than employing cognitive interviews for the entire survey, we adopted a more pragmatic approach, where the surveys were discussed, and staff were encouraged to highlight any difficulties they encountered. Two researchers participated in both the interviews and the subsequent analysis, ensuring methodological rigor. |
| **Sample size** | | |  |
| Gathering enough data from the right people (Boateng et al. 2018); Not an independent entity in Mokkink et al. 2017 | 4.1 Administer potential scale items on a sample that reflects range of target population using paper or device | 1.5. Was the [survey] development study performed in a sample representing the target population for which the [survey] was developed? | The sample size (> 400 respondents) was sufficient and encompassed the target population. |
|  | 4.2 Recommended sample size is 10 respondents per survey item and/or 200-300 observations | 3.3 Was the sample size included in the analysis adequate? (at least 5 times the number of items and ≥100; OR at least 6 times number of items but <100) |  |
|  | 4.3 Use cross-sectional data for exploratory factor analysis | NA | We used a cross-sectional study design, not employ a longitudinal dataset. |
|  | 4.4 Use data from a second time point, at least 3 months later in a longitudinal dataset, or an independent sample for test of dimensionality | NA |  |
| **Structural validity and tests of dimensionality** | | |  |
| Structural validity (Mokkink et al. 2017); Ensuring that scale is parsimonious, extraction of factors and tests of dimensionality (Boateng et al. 2018) | 5.1 Determine the proportion of correct answers given per item using Clasical Test THeory (CTT). Determine the probability of a particular examinee correctly answering a given item - using Item Response Theory (IRT). 5.2 Estimate biserial correlations or item discrimination parameter using statistical packages 5.3 Estimate inter-item/item communalities, item-total, and adjusted item-total correlations using statistical packages 5.4 Estimate distractor analysis using statistical packages 6.1 Use scree plots, exploratory factor analysis, parallel analysis, minimum average partial procedure, and/or the Hull method 7.1 To validate whether the previous hypothetical structure fits–the items - Estimate independent cluster model—confirmatory factor analysis.  7.2 Estimate bifactor models to eliminate ambiguity about the type of dimensionality, unidimensionality, bidimensionality, or multi-dimensionality 7.3 Estimate measurement invariance to determine whether hypothesized factor and dimension is congruent across groups or multiple samples | 3.1 For CTT: Was exploratory or confirmatory factor analysis performed? (confirmatory factor analysis preferred) 3.2 For IRT/Rasch: does the chosen model fit to the research question? 3.4 Were there any other important flaws in the design or statistical methods of the study? | To assess structural validity, we applied Classical Test Theory, specifically Confirmatory Factor Analysis (CFA), conducting both first- and second-order CFA to clarify the dimensional structure. The analysis included factor loadings, variances, covariances, and residual error variances, with no identified methodological flaws.  Although Item Response Theory (IRT) and Rasch models were within scope, they were not employed, as our primary aim was to evaluate the factorial structure rather than item-level properties such as discrimination and difficulty. IRT is particularly useful for analyzing item functioning across ability levels, whereas our focus was on scale-level validity. While a combined CTT-IRT approach could have been possible, CFA was prioritized due to its interpretability and suitability for our objectives.  Given that we conducted a second-order CFA to examine the hierarchical structure, bifactor models were not estimated, as both approaches address similar dimensionality questions. Measurement invariance was not tested, as the study did not aim to compare latent structures across distinct groups.  Distractor analysis was not applicable, as it is only relevant for multiple-choice formats. |
| **Scale scores** | | |  |
| Create scale scores for substantive analysis including reliability and validity of scale (Boateng et al. 2018);  Not described in Mokkink et al. 2017 | 7.4 Calculate scale scores using an unweighted approach, which includes summing standardized item scores and raw item scores, or computing the mean for raw item scores 7.5 Calculate scale scores by using a weighted approach, which includes creating factor scores via confirmatory factor analysis or structural equation models | NA | Given our study's objectives, we prioritized evaluating the factorial structure rather than generating composite scores for applied use. |
| **Reliability and internal consistency** | | |  |
| Reliability (Boateng et al. 2018);  Internal consistency and reliability (Mokkink et al. 2017) | 8.1 Estimate using Cronbach’s alpha  8.2. Other tests such as Raykov’s rho, ordinal alpha, and Revelle’s beta can be used to assess scale reliability  8.3 Estimate the strength of the relationship between scale items over two or three time points; variety of measures possible | 4.1 Was an internal consistency statistic calculated for each unidimensional scale or subscale separately?  4.2 For continuous scores: Was Cronbach’s alpha or  omega calculated?  4.3 For dichotomous scores: Was Cronbach’s alpha or KR‐  20 calculated?  4.4 For IRT‐based scores: Was standard error of the theta  (SE (θ)) or reliability coefficient of estimated latent  trait value (index of (subject or item) separation)  calculated?  4.5 Were there any other important flaws in the design  or statistical methods of the study? | We assessed **internal consistency** by calculating **Cronbach’s alpha and McDonald’s omega** for each unidimensional scale and subscale separately (guidelines 4.1, 4.2, 8.1). These measures are appropriate for evaluating the reliability of continuous scale scores.  Alternative reliability indices such as **Raykov’s rho, ordinal alpha, and Revelle’s beta** (guideline 8.2) were not calculated, as alpha and omega were deemed sufficient for assessing internal consistency in this study.  We did not estimate the **strength of relationships between scale items over multiple time points** (guideline 8.3), as the study was cross-sectional and did not include repeated measurements.  Since our data were **continuous**, KR-20 (guideline 4.3) was not relevant. We also did not apply **IRT-based reliability measures** such as the standard error of theta or item/subject separation indices (guideline 4.4), as our analysis followed a Classical Test Theory approach rather than an IRT-based framework. |
|  | NA | *Note: Box 5 "cross-cultural validity" is not included in this table.* | Not applicable for this study as it concerns “the degree to which the performance of the items on a translated or culturally adapted outcome measure are an adequate reflection of the performance of the items of the original version of the outcome measure” [36]. |
|  | NA | 6.1 Were patients stable in the interim period on the construct to be measured? 6.2 Was the time interval appropriate? 6.3 Were the test conditions similar for the measurements? e.g. type of administration, environment, instructions 6.4 For continuous scores: Was an intraclass correlation coefficient (ICC) calculated? 6.5 For dichotomous/nominal/ordinal scores: Was kappa calculated?  6.6 For ordinal scores: Was a weighted kappa calculated? 6.7 For ordinal scores: Was the weighting scheme Described? e.g. linear, quadratic 6.8 Were there any other important flaws in the design or statistical methods of the study | To assess inter-rater reliability, we calculated the intraclass correlation coefficient (ICC) – guideline 6.4. ICC was chosen as it is the appropriate measure for evaluating agreement between raters when ratings are continuous. A potential methodological limitation in using ICC is that it assumes stability in the construct being measured, yet no repeated measurement was conducted to confirm this (guidelines 6.1, 7.1).  Since the study was cross-sectional, temporal stability was not assessed, as there was no interim period between measurements. Consequently, the appropriateness of the time interval (guidelines 6.2, 7.2) and consistency of test conditions across multiple measurement points (guidelines 6.3, 7.3) were not applicable.  As data were collected via an electronic questionnaire, we did not control the administration environment (guidelines 6.3, 7.3), but this is standard practice for survey-based research in school settings.  We did not calculate Standard Error of Measurement (SEM), Smallest Detectable Change (SDC), or Limits of Agreement (LoA) (guideline 7.4), as our focus was on inter-rater reliability rather than measurement precision at the individual level. Similarly, kappa statistics for dichotomous, nominal, or ordinal data (guidelines 6.5, 6.6, 6.7, 7.5) were not relevant, as our data were continuous.  We did not conduct additional validity analyses such as correlations or the area under the receiver operating curve (AUC) (guideline 8.1). Sensitivity and specificity (guideline 8.2) were not assessed, as they are primarily relevant for evaluating a test’s ability to classify individuals into binary categories, which was not the aim of this study.  Furthermore, our target population consisted of school staff, not patients, making certain stability-related considerations from clinical settings less relevant (guidelines 6.1, 7.1).  Potential methodological limitations include the assumption of stability in ICC calculations and the cross-sectional nature of the study (guidelines 6.8, 7.6, 8.3). |
| **Measurement error and criterion validity** | |  |  |
| Measurement error and criterion validity (Mokkink et al. 2017);  Not described in Boateng et al. 2018 | NA | 7.1 Were patients stable in the interim period on the construct to be measured? 7.2 Was the time interval appropriate?  7.3 Were the test conditions similar for the measurements? (e.g. type of administration, environment, instructions) 7.4 For continuous scores: Was the Standard Error of Measurement (SEM), Smallest Detectable Change (SDC) or Limits of Agreement (LoA) calculated  7.5 For dichotomous/nominal/ordinal scores: Was the percentage (positive and negative) agreement calculated?  7.6 Were there any other important flaws in the design or statistical methods of the study? |  |
|  |  | 8.1 For continuous scores: Were correlations, or the area under the receiver operating curve calculated?  8.2 For dichotomous scores: Were sensitivity and specificity determined?  8.3 Were there any other important flaws in the design or statistical methods of the study? |  |
| **Construct validity (Concurrent validity, Convergent validity, Discriminant validity, Known-groups validity, Correlation analysis, construct approach)** | | |  |
| Concurrent validity (Boateng et al. 2018);  Criterion approach (Mokkink et al. 2017) | 9.2 To determine the extent to which scale scores have a stronger relationship with criterion measurements made near the time of administration.  Estimate the association between scale scores and “gold standard” of scale measurement; stronger significant association in Pearson product-moment correlation suggests support for concurrent validity | Comparison to a gold standard. 10.1 For continuous scores: Were correlations between change scores, or the area under the Receiver Operator Curve (ROC) curve calculated? 10.2 For dichotomous scales: Were sensitivity and specificity (changed versus not changed) determined? 10.3 Were there any other important flaws in the design or statistical methods of the study? | Not applicable. We did not assess concurrent validity (guideline 9.2) due to both practical constraints and the lack of an established gold standard in this field. Including an alternative measure (e.g., HPS) would have complicated the survey structure and increased respondent burden without necessarily improving validity assessment.  Similarly, we did not calculate correlations between change scores or the area under the ROC curve (guideline 10.1), as the study was cross-sectional. Sensitivity and specificity (guideline 10.2) were also not applicable, as we did not assess change over time. |
| Convergent validity (Boateng et al. 2018; Mokkink et al. 2017) | To examine if the same concept measured in different ways yields similar results (convergent validity) 9.3 Estimate the relationship between scale scores and similar constructs using multi-trait multi-method matrix, latent variable modeling, or Pearson product-moment coefficient; higher/stronger correlation coefficients suggest support for convergent validity | 9a Comparison with other outcome measurement instruments (convergent validity) 9.1 Is it clear what the comparator instrument(s) measure(s) 9.2 Were the measurement properties of the comparator instrument(s) sufficient? 9.3 Was the statistical appropriate for the hypotheses to be tested? 9.4 Were there any other important flaws in the design or statistical methods of the study? | To assess convergent and discriminant validity, we applied a multi-trait multi-method (MTMM) approach, specifically following Widaman's (1985) framework. This method allowed us to examine the relationship between the two included scales, HPS and WSA-PA, to determine the extent to which they measure related but distinct constructs.  While this approach aligns with recommended statistical methods for evaluating convergent and discriminant validity, there were some limitations. Due to the small number of items in certain sub-factors, we had to exclude some subscale comparisons, which may have constrained the full assessment of convergent and discrimant validity.  We did not conduct additional analyses for known-groups validity or comparisons with external outcome measurement instruments beyond HPS and WSA-PA. Additionally, we did not perform statistical subgroup comparisons, as our focus was on scale-level relationships rather than subgroup differences.  Despite these limitations, our approach adheres to the core recommendations for assessing convergent and discriminant validity through an established MTMM-framework. |
| Discriminant validity and known groups validity (Boateng et al. 2018);  Comparison between subgroups (Mokkink et al. 2017) | To examine if the concept measured is different from some other concept (discriminant validity)  9.4 Estimate the relationship between scale scores and distinct constructs using multi-trait multi-method matrix, latent variable modeling, or Pearson product-moment coefficient; lower/weaker correlation coefficients suggest support for discriminant validity To examine if the concept measured behaves as expected in relation to “known groups” 9.5 Select known binary variables based on theoretical and empirical knowledge and determine the distribution of the scale scores over the known groups; use t-tests if binary, ANOVA if multiple groups | Comparison between subgroups (discriminative or known‐groups validity) 9.5 Was an adequate description provided of important characteristics of the subgroups? 9.6 Was the statistical method appropriate for the hypotheses to be tested? 9.7 Were there any other important flaws in the design or statistical methods of the study? |  |
| Correlation analysis (Boateng et al. 2018) | To determine the relationship between existing measures or variables and newly developed scale scores 9.6 Correlate scale scores and existing measures or variables, preferably, use linear regression, intraclass correlation coefficient, and analysis of standard deviations of the differences between scores | NA | We did not yet examine the relationship between scale scores and external measures using linear regression, intraclass correlation coefficient, or standard deviation analysis. This will be explored in future analyses when assessing associations between the validated scales and relevant factors. |
| Construct approach (Mokkink et al. 2017) | NA | Hypotheses testing; comparison with other outcome measurement instruments. 10.4 Is it clear what the comparator instrument(s) measure(s)? 10.5 Were the measurement properties of the comparator instrument(s) sufficient? 10.6 Was the statistical method appropriate for the hypotheses to be tested? 10.7 Were there any other important flaws in the design or statistical methods of the study? Hypotheses testing: comparison between subgroups 10.8 Was an adequate description provided of important characteristics of the subgroups? 10.9 Was the statistical method appropriate for the hypotheses to be tested? 10.10 Were there any other important flaws in the design or statistical methods of the study? Hypotheses testing: before and after intervention) 10.11 Was an adequate description provided of the intervention given? 10.12 Was the statistical method appropriate for the hypotheses to be tested? 10.13 Were there any other important flaws in the design or statistical methods of the study? | We did not perform hypothesis testing involving comparisons with other outcome measurement instruments (guidelines 10.4–10.7), as no additional comparator instruments beyond HPS and WSA-PA were included in the study.  We did not conduct subgroup comparisons (guidelines 10.8–10.10), as the study did not aim to test differences between predefined subgroups.  Hypothesis testing related to before-and-after intervention effects (guidelines 10.11–10.13) was **not applicable**, as this study did not involve an intervention. |

**Appendix 2: Guidelines for using the Health Promoting Schools (HPS) Questionnaire**

***This appendix provides detailed instructions for researchers and practitioners interested in utilizing the Health Promoting Schools (HPS) questionnaire.***

Thank you for your interest in the HPS questionnaire. This instrument was developed by the Department for Prevention, Health Promotion, and Community Care at Steno Diabetes Center Copenhagen for use at Preparatory Basic Education and Training Schools serving NEET (Not in Employment, Education, or Training) youth in Denmark. It is adaptable for use in primary, secondary, vocational, and special needs schools, with appropriate modifications to suit the specific context (e.g., school type, country, and target population). It is free of use but please acknowledge the source by referencing this article if used.

The questionnaire consists of 24 items designed to assess the implementation level of seven distinct subfactors, which collectively provide a total HPS score reflecting the school’s overall approach to student health and well-being. In addition to this overall indication, the subfactors provide targeted insights into specific implementation areas, enabling schools to identify strengths and areas for improvement. It is intended to be completed by all staff members who have regular contact with students.

Please note that the questionnaire has not been validated in English.

**Questionnaire Structure**

The questionnaire is organized into seven subdomains:

1. School policies
2. School ethos
3. Collaboration and involvement
4. School practice
5. Quality of delivery
6. Physical and financial resources
7. School health services

Each item is answered using a single response option. The questionnaire was initially developed for online administration, and online data collection is therefore recommended.

**Modifications to the Questionnaire**

To optimize alignment with specific HPS programs, the following adjustments may be made without significantly compromising the validity or psychometric properties:

- Customizing references to the specific HPS program being evaluated.
- Adding, removing, or rephrasing health promotion topics (applicable for items 1, 12, 13, 15).
- Adapting terms to align with the specific context or sector.

**Scoring System**

The questionnaire produces seven distinct scores, one for each subfactor, which together reflect different dimensions of a school's health-promoting practices. These sub-scores provide actionable insights for policy and practice. Additionally, a total HPS score can be calculated by first averaging the items within each subfactor to obtain subfactor scores and then averaging these subfactor scores to derive the total score.

**General Recoding Instructions:**

- Recode “Don’t know” responses as missing values and exclude them from scoring.

**Subfactor Recoding Instructions:**

1. **Subfactor 1:** Count the number of “Yes” responses across the 8 health themes. Recode the total sum into one variable with the following categories:
   - 1: No policies
   - 2: Policies within 1–2 themes
   - 3: Policies within 3–4 themes
   - 4: Policies within 5–6 themes
   - 5: Policies within 7–8 themes
2. **Subfactor 2:** Calculate the mean score across Items 2–6.
3. **Subfactor 3:** Calculate the mean score across Items 7–11.
4. **Subfactor 4:** Recode the six response categories into the following five:
   - 1: Never/less frequently
   - 2: About 1–3 times a month
   - 3: About once a week
   - 4: Almost daily/several times a week
   - 5: Daily/multiple times a day

Compute mean scores for Items 12a–h and 13a–h separately, then average these to generate a total score for Subfactor 4.

1. **Subfactor 5:** Compute the mean score for Items 15a–15h and then for all Items 14–19.
2. **Subfactor 6:** Calculate the mean score across Items 20–23.
3. **Subfactor 7:** Use the single-item score from Item 22 as the subfactor score.

**Health Promoting School (HPS) Questionnaire. English Version.**

| SUBFACTOR 1: SCHOOL POLICIES |
| --- |

| 1. Does the school have a written policy, vision or action plan that covers one or more of the following areas? The policy, vision or action plan may be written on the school's website or in the school rules.  If you are unsure, pick "don't know". | | | |
| --- | --- | --- | --- |
|  | **Yes** (1) | **No** (2) | **Don’t know** (0) |
| 1a. Physical activity | 🔾 | 🔾 | 🔾 |
| 1b. Food and meals | 🔾 | 🔾 | 🔾 |
| 1c. Mental health (e.g., loneliness, performance-related anxiety, and stress) | 🔾 | 🔾 | 🔾 |
| 1d. Social relations | 🔾 | 🔾 | 🔾 |
| 1e. Gender, body and sexuality | 🔾 | 🔾 | 🔾 |
| 1f. Smoking and nicotine products | 🔾 | 🔾 | 🔾 |
| 1g. Alcohol and drugs | 🔾 | 🔾 | 🔾 |
| 1h. Sleep and tiredness | 🔾 | 🔾 | 🔾 |

| 2. Promoting students’ health and well-being is part of the school’s core values. |
| --- |

| 3. The school is responsible for promoting health and well-being among students. |
| --- |

| 4. The school has zero tolerance towards bullying/online bullying and discrimination. |
| --- |

| 5. The school staff's practice is based on the understanding that there is a link between students' learning and their health and well-being.   \| 6. The management sets goals and direction for the school’s work with health and well-being.  Response scale for items no. 2-6:   \| Very high extent  (5) \| High extent  (4) \| Neutral  (3) \| Low extent  (2) \| Not at all  (1) \| Don’t know  (0) \| \| --- \| --- \| --- \| --- \| --- \| --- \| \| \| --- \| --- \| --- \| --- \| --- \| --- \| --- \| \|  \| |
| --- | --- | --- | --- | --- | --- | --- | --- | --- |

| SUBFACTOR 2: SCHOOL ETHOS |
| --- |

| SUBFACTOR 3: COLLABORATION AND INVOLVEMENT |
| --- |

| 7. Are staff involved in the school's efforts to decide on or develop its practice regarding health and well-being? |
| --- |
| 8. Are students involved in the school's efforts to decide on or develop its practices regarding health and well-being? |
| 9. Does the school collaborate with external stakeholders on individual-targeted initiatives regarding health and well-being? Examples of individualized interventions include therapy, substance abuse support, dietitian services, or other forms of personalized guidance. |
| 10. Does the school collaborate with external stakeholders on other than individual-targeted initiatives regarding health and well-being? Examples of other than individual-targeted initiatives include development of health policies, competence development of school staff or initiatives targeted groups of students. Moreover, if external stakeholders help the school organizing events, theme days, or teaching activities. |
| 11. School practices, including teaching and other activities, are based on active participation and involvement of students. |

**Response scale for items no. 7-11:**

| Very high extent  (5) | High extent  (4) | Neutral  (3) | Low extent  (2) | Not at all  (1) | Don’t know  (0) |
| --- | --- | --- | --- | --- | --- |

| SUBFACTOR 4: SCHOOL PRACTICE |
| --- |

| 12. How often do you work with organized or planned activities within the following areas during the school day? Consider both practical and theoretical activities, as well as activities during breaks and special events. These activities may be organized by you or in collaboration with external partners. |
| --- |

| 13. How often do you have informal conversations with students about the following topics during the school day? |
| --- |

**Areas and response scales for items no. 12-13:**

|  | Daily or multiple times a day (6) | Almost daily/several times a week (5) | About once a week (4) | About one to three times a month (3) | Less frequently (2) | Never (1) | Don’t know (0) |
| --- | --- | --- | --- | --- | --- | --- | --- |
| 12a, 13a. Physical activity | 🔾 | 🔾 | 🔾 | 🔾 | 🔾 | 🔾 | 🔾 |
| 12b, 13b. Food and meals | 🔾 | 🔾 | 🔾 | 🔾 | 🔾 | 🔾 | 🔾 |
| 12c, 13c. Mental health | 🔾 | 🔾 | 🔾 | 🔾 | 🔾 | 🔾 | 🔾 |
| 12d, 13d. Social relations | 🔾 | 🔾 | 🔾 | 🔾 | 🔾 | 🔾 | 🔾 |
| 12e, 13e. Gender, body and sexually | 🔾 | 🔾 | 🔾 | 🔾 | 🔾 | 🔾 | 🔾 |
| 12f, 13f. Smoking and nicotine products | 🔾 | 🔾 | 🔾 | 🔾 | 🔾 | 🔾 | 🔾 |
| 12g, 13g. Alcohol and drugs | 🔾 | 🔾 | 🔾 | 🔾 | 🔾 | 🔾 | 🔾 |
| 12h, 13h. Sleep and tiredness | 🔾 | 🔾 | 🔾 | 🔾 | 🔾 | 🔾 | 🔾 |
|  |  |  |  |  |  |  |  |

| SUBFACTOR 5: QUALITY OF DELIVERY |
| --- |

| 14. I have sufficient skills and knowledge to work with health and well-being. | | | | | | | |
| --- | --- | --- | --- | --- | --- | --- | --- |
| 15. Are you motivated for working with health and well-being within… | | | | | | |  |
| 15a. Physical activity | 🔾 | 🔾 | 🔾 | 🔾 | 🔾 | 🔾 |  |
| 15b. Food and meals | 🔾 | 🔾 | 🔾 | 🔾 | 🔾 | 🔾 |  |
| 15c. Mental health (e.g., loneliness, performance-related anxiety, and stress) | 🔾 | 🔾 | 🔾 | 🔾 | 🔾 | 🔾 |  |
| 15d. Social relations | 🔾 | 🔾 | 🔾 | 🔾 | 🔾 | 🔾 |  |
| 15e. Gender, body and sexuality | 🔾 | 🔾 | 🔾 | 🔾 | 🔾 | 🔾 |  |
| 15f. Smoking and nicotine products | 🔾 | 🔾 | 🔾 | 🔾 | 🔾 | 🔾 |  |
| 15g. Alcohol and drugs | 🔾 | 🔾 | 🔾 | 🔾 | 🔾 | 🔾 |  |
| 15h. Sleep and tiredness | 🔾 | 🔾 | 🔾 | 🔾 | 🔾 | 🔾 |  |

| 16. To what extent is your practice regarding health and well-being based on the best available knowledge (e.g., recommendations from the national health authority or local government)? |
| --- |
| 17. To what extent is your practice regarding health and well-being based on local knowledge and needs (e.g., requests from staff, student councils, or data from student surveys). |
| 18. In your daily work, to what extent do you seek to increase students’ action competence and health literacy - i.e., their ability to access, understand, and act on information related to health and well-being? |
| 19. To what extent do you encourage students to participate in leisure time activities (e.g., sports, creative activities, or volunteer work)? |

| Very high extent  (5) | High extent  (4) | Neutral  (3) | Low extent  (2) | Not at all  (1) | Don’t know  (0) |
| --- | --- | --- | --- | --- | --- |

**Response scale for items no. 14-19:**

| SUBFACTOR 6: PHYSICAL AND FINANCIAL RESOURCES |
| --- |

| 20. The school’s physical environment is suitable for working with health and well-being (e.g., outdoor areas, premises). |
| --- |
| 21. The school has the financial resources needed for working with student’s health and well-being (e.g., to start up new initiatives). |
| 22. The management prioritizes competence development regarding health and well-being (e.g., by enabling staff to participate in courses). |
| 23. I have sufficient time to work on students’ health and well-being. |

| Very high extent  (5) | High extent  (4) | Neutral  (3) | Low extent  (2) | Not at all  (1) | Don’t know  (0) |
| --- | --- | --- | --- | --- | --- |

**Response scale for items no. 20-23:**

| SUBFACTOR 7: SCHOOL HEALTH SERVICES |
| --- |

| 24. Do you know whom you can refer students to if they experience health or well-being-related issues (e.g., someone connected to the school or an external professional)?   \| Very high extent  (5) \| High extent  (4) \| Neutral  (3) \| Low extent  (2) \| Not at all  (1) \| Don’t know  (0) \| \| --- \| --- \| --- \| --- \| --- \| --- \|   Response scale for item no. 24: |
| --- | --- | --- | --- | --- | --- | --- |

**Health Promoting School (HPS) Questionnaire. Danish version**

| SUBFAKTOR 1: POLITIKKER |
| --- |

| 1. Har skolen en skriftlig politik, vision eller handleplan, der indeholder et eller flere af følgende områder? Politikken, visionen eller handleplanen kan fx være beskrevet på skolens hjemmeside eller i skolens ordensregler. Tænk på skolens politikker, visioner eller handleplaner udover FGU's didaktiske princip om sundhed, ernæring og motion samt formålsparagraffer, der gælder for alle FGU-institutioner.  Hvis du er i tvivl, skal du vælge ”ved ikke”. | | | |
| --- | --- | --- | --- |
|  | **Ja** (1) | **Nej** (2) | **Ved ikke** (0) |
| 1a. Motion og bevægelse | 🔾 | 🔾 | 🔾 |
| 1b. Mad og måltider | 🔾 | 🔾 | 🔾 |
| 1c. Psykisk (mis)trivsel (fx ensomhed, præstationsangst og stress) | 🔾 | 🔾 | 🔾 |
| 1d. Fællesskaber og sociale relationer | 🔾 | 🔾 | 🔾 |
| 1e. Køn, krop og seksualitet | 🔾 | 🔾 | 🔾 |
| 1f. Rygning og nikotin produkter | 🔾 | 🔾 | 🔾 |
| 1g. Alkohol og stoffer | 🔾 | 🔾 | 🔾 |
| 1h. Søvn og træthed | 🔾 | 🔾 | 🔾 |
|  |  |  |  |

| SUBFAKTOR 2: KERNEVÆRDIER |
| --- |

| 2. Det er en del af skolens kerneværdier at fremme elevernes sundhed og trivsel. |
| --- |
| 3. Det er skolens ansvar at bidrage til at fremme elevernes sundhed og trivsel. |
| 4. Skolen har nultolerance overfor mobning/online mobning og diskrimination. |
| 5. Skolens ansatte arbejder ud fra en forståelse af, at der er en sammenhæng mellem elevernes læring og deres sundhed og trivsel. |
| 6. Ledelsen sætter mål og retning for skolens arbejde inden for sundhed og trivsel. |

| Svarkategorier til spørgsmål nr. 2-6: | | | | | |
| --- | --- | --- | --- | --- | --- |
| I meget høj grad  (5) | I høj grad  (4) | Hverken i høj eller lav grad  (3) | I lav grad  (2) | Slet ikke  (1) | Ved ikke  (0) |
|  |  |  |  |  |  |

| SUBFAKTOR 3: SAMARBEJDE OG INVOLVERING |
| --- |

| 7. Bliver skolens ansatte involveret i skolens arbejde med at beslutte eller udvikle skolens praksis inden for sundhed og trivsel?? | | | | | |
| --- | --- | --- | --- | --- | --- |
| 8. Bliver skolens elever involveret i skolens arbejde med at beslutte eller udvikle skolens praksis inden for sundhed og trivsel??? | | | | | |
| 9. Samarbejder skolen med eksterne aktører om individrettede indsatser inden for sundhed og trivsel? Individrettede indsatser kan fx være psykologhjælp, misbrugshjælp, diætist eller andre former for individuel vejledning. udsagn? | | | | | |
| 10. Samarbejder skolen med eksterne aktører om andet end individrettede indsatser inden for sundhed og trivsel? Det kan fx være udvikling af en sundhedspolitik, kompetenceudvikling af medarbejdere eller indsatser målrettet grupper eller hold af elever. Det kan også være, hvis eksterne hjælper med at afholde events, temadage eller undervisningsaktiviteter.? | | | | | |
| 11. Skolens praksis, herunder undervisning og andre aktiviteter, er baseret på aktiv deltagelse og involvering af eleverne.  Svarkategorier til spørgsmål nr. 7-11: | | | | | |
| I meget høj grad  (5) | I høj grad  (4) | Hverken i høj eller lav grad  (3) | I lav grad  (2) | Slet ikke  (1) | Ved ikke  (0) |
|  |  |  |  |  |  |

| SUBFAKTOR 4: SKOLEPRAKSIS |
| --- |

| 12. Hvor ofte arbejder du med organiserede og planlagte aktiviteter inden for følgende områder i løbet af skoledagen? Tænk både på praktiske eller teoretiske undervisningsaktiviteter, aktiviteter i pauser og i forbindelse med særlige begivenheder (fx events eller temadage). Det kan være noget du selv gør eller i samarbejde med eksterne aktører.  13. Hvor ofte har du uformelle samtaler med elever om følgende områder i løbet af skoledagen?  Områder og svarkategorier for spørgsmål nr. 12-13: | | | | | | | |
| --- | --- | --- | --- | --- | --- | --- | --- |
|  | Dagligt eller flere gange dagligt  (6) | Næsten dagligt/ flere gange om ugen  (5) | Ca. en gang om ugen  (4) | Ca. en-tre gange om måneden  (3) | Sjældnere  (2) | Aldrig  (1) | Ved ikke  (0) |
| 12a, 13a Motion og bevægelse | 🔾 | 🔾 | 🔾 | 🔾 | 🔾 | 🔾 | 🔾 |
| 12b, 13b Mad og måltider | 🔾 | 🔾 | 🔾 | 🔾 | 🔾 | 🔾 | 🔾 |
| 12c, 13c Psykisk (mis)trivsel | 🔾 | 🔾 | 🔾 | 🔾 | 🔾 | 🔾 | 🔾 |
| 12d, 13d Fællesskaber og sociale relationer | 🔾 | 🔾 | 🔾 | 🔾 | 🔾 | 🔾 | 🔾 |
| 12e, 13e Køn, krop og seksualitet | 🔾 | 🔾 | 🔾 | 🔾 | 🔾 | 🔾 | 🔾 |
| 12f, 13f Rygning og nikotin produkter | 🔾 | 🔾 | 🔾 | 🔾 | 🔾 | 🔾 | 🔾 |
| 12g, 13g Alkohol og stoffer | 🔾 | 🔾 | 🔾 | 🔾 | 🔾 | 🔾 | 🔾 |
| 12h, 13h Søvn og træthed | 🔾 | 🔾 | 🔾 | 🔾 | 🔾 | 🔾 | 🔾 |

| SUBFAKTOR 5: KVALITET I PRAKSIS |
| --- |

| 14. Jeg har tilstrækkelig viden og kompetencer til at arbejde med sundhed og trivsel. | | | | | | | |
| --- | --- | --- | --- | --- | --- | --- | --- |
| 15. Er du motiveret for at arbejde med tiltag inden for… | | | | | | |  |
| 15a. Motion og bevægelse | 🔾 | 🔾 | 🔾 | 🔾 | 🔾 | 🔾 |  |
| 15b. Mad og måltider | 🔾 | 🔾 | 🔾 | 🔾 | 🔾 | 🔾 |  |
| 15c. Psykisk (mis)trivsel (fx ensomhed, præstationsangst og stress) | 🔾 | 🔾 | 🔾 | 🔾 | 🔾 | 🔾 |  |
| 15d. Fællesskaber og sociale relationer | 🔾 | 🔾 | 🔾 | 🔾 | 🔾 | 🔾 |  |
| 15e. Køn, krop og seksualitet | 🔾 | 🔾 | 🔾 | 🔾 | 🔾 | 🔾 |  |
| 15f. Rygning og nikotinprodukter | 🔾 | 🔾 | 🔾 | 🔾 | 🔾 | 🔾 |  |
| 15g. Alkohol og stoffer | 🔾 | 🔾 | 🔾 | 🔾 | 🔾 | 🔾 |  |
| 15h. Søvn og træthed | 🔾 | 🔾 | 🔾 | 🔾 | 🔾 | 🔾 |  |

| 16. Vurderer du, at din praksis inden for sundhed og trivsel er baseret på den bedst tilgængelige viden (det kan fx være anbefalinger fra Sundhedsstyrelsen eller kommunen)? | | | | | |
| --- | --- | --- | --- | --- | --- |
| 17. Vurderer du, at din praksis inden for sundhed og trivsel er baseret på lokal viden og behov (det kan fx være ønsker fra medarbejdere, elevråd eller data fra trivselsmålinger)? | | | | | |
| 18. Har du i dit arbejde fokus på at øge elevernes handle- og sundhedskompetence, dvs. elevernes evne til at tilgå, forstå og handle på information i forhold til sundhed og trivsel? | | | | | |
| 19. Opfordrer du elever til at have et aktivt fritidsliv? Det kan fx være at deltage i sport, kreative aktiviteter, musik eller frivilligt arbejde i deres fritid. | | | | | |
|  | | | | | |
| Svarkategorier til spørgsmål nr. 14-19: | | | | | |
| I meget høj grad  (5) | I høj grad  (4) | Hverken i høj eller lav grad  (3) | I lav grad  (2) | Slet ikke  (1) | Ved ikke  (0) |

| SUBFAKTOR 6: FYSISK MILJØ OG RESSOURCER |
| --- |

| 20. Skolens fysiske rammer er egnede til at arbejde med sundhed og trivsel (fx udendørsarealer, lokaler). | | | | | |
| --- | --- | --- | --- | --- | --- |
| 21. Skolen har et økonomisk råderum, der gør det muligt at arbejde med elevernes sundhed og trivsel (fx igangsætte nye tiltag). | | | | | |
| 22. Ledelsen prioriterer kompetenceudvikling inden for sundhed og trivsel (fx ved at ansatte får mulighed for at deltage i kurser). | | | | | |
| 23. Jeg har tilstrækkelig tid til at arbejde med sundhed og trivsel. | | | | | |
| Svarkategorier til spørgsmål nr. 20-23: | | | | | |
| I meget høj grad  (5) | I høj grad  (4) | Hverken i høj eller lav grad  (3) | I lav grad  (2) | Slet ikke  (1) | Ved ikke  (0) |
|  |  |  |  |  |  |

| SUBFAKTOR 7: SUNDHEDSFAGLIGT PERSONALE |
| --- |

| 24. Ved du, hvem du kan henvise elever til, hvis de oplever sundheds- eller trivselsrelaterede udfordringer? Det kan både være en person på skolen eller en person/organisation udenfor skolen. | | | | | |
| --- | --- | --- | --- | --- | --- |
| Svarkategorier til spørgsmål nr. 24: | | | | | |
| I meget høj grad  (5) | I høj grad  (4) | Hverken i høj eller lav grad  (3) | I lav grad  (2) | Slet ikke  (1) | Ved ikke  (0) |

**Appendix 3: Guidelines for using the Whole School approach to Physical Activity (WSA-PA) Questionnaire**

***This appendix provides detailed instructions for researchers and practitioners interested in utilizing the Whole School approach to Physical Activity (WSA-PA) questionnaire.***

Thank you for your interest in the WSA-PA questionnaire. This instrument was developed by the Department for Prevention, Health Promotion, and Community Care at Steno Diabetes Center Copenhagen for use at Preparatory Basic Education and Training Schools serving NEET (Not in Employment, Education, or Training) youth in Denmark. It is adaptable for use in primary, secondary, vocational, and special needs schools, with appropriate modifications to suit the specific context (e.g., school type, country, and target population). It is free of use but please acknowledge the source by referencing this article if used.

The questionnaire consists of 21 items designed to assess the implementation level of six distinct subfactors, which collectively provide a total WSA-PA score reflecting the school’s overall approach to student physical activity. In addition to this overall indication, the subfactors provide targeted insights into specific implementation areas, enabling schools to identify strengths and areas for improvement. It is intended to be completed by all staff members who have regular contact with students.

Please note that the questionnaire has not been validated in English.

**Questionnaire Structure**

The questionnaire is organized into six subdomains:

1. School ethos
2. Collaboration and involvement
3. School practice
4. Quality of delivery
5. Physical and financial resources
6. School health services

Each item is answered using a single response option. The questionnaire was initially developed for online administration, and online data collection is therefore recommended.

**Modifications to the Questionnaire**

To optimize alignment with specific WSA-PA programs, the following adjustments may be made without significantly compromising the validity or psychometric properties:

- Customizing references to the specific WSA-PA program being evaluated.
- Adapting terms to align with the specific context or sector.

**Scoring System**

The questionnaire produces six distinct scores, one for each subfactor, which together reflect different dimensions of a school's WSA-PA. These sub-scores provide actionable insights for policy and practice. Additionally, a total WSA-PA score can be calculated by first averaging the items within each subfactor to obtain subfactor scores and then averaging these subfactor scores to derive the total score.

**General Recoding Instructions:**

- Recode “Don’t know” responses as missing values and exclude them from scoring.

**Subfactor Recoding Instructions:**

1. **Subfactor 1:** Calculate the mean score across Items 1–4.
2. **Subfactor 2:** Calculate the mean score across Items 5-8.
3. **Subfactor 3:** Recode the six response categories for items 9-10 into the following five:
   - 1: Never/less frequently
   - 2: About 1–3 times a month
   - 3: About once a week
   - 4: Almost daily/several times a week
   - 5: Daily/multiple times a day

Compute scores for Items 9 and 10 based and calculate the mean score across Item 9–10.

1. **Subfactor 4:** Calculate the mean score across Items 11–16.
2. **Subfactor 5:** Calculate the mean score across Items 17–20.
3. **Subfactor 6:** Use the single-item score from Item 21 as the subfactor score.

**Whole School approach to Physical Activity (WSA-PA) Questionnaire. English Version.**

| SUBFACTOR 1: SCHOOL ETHOS |
| --- |

| 1. Promoting students’ physical activity is part of the school’s core values. |
| --- |
| 2. The school is responsible for promoting physical activity among students. |
| 3. The school staff's practice is based on the understanding that there is a link between students' learning and their physical activity. |
| 4. The management sets goals and direction for the school’s work with physical activity. |

**Response scale for items no. 1-4:**

| Very high extent  (5) | High extent  (4) | Neutral  (3) | Low extent  (2) | Not at all  (1) | Don’t know  (0) |
| --- | --- | --- | --- | --- | --- |

| SUBFACTOR 2: COLLABORATION AND INVOLVEMENT |
| --- |

| 5. Staff are involved in deciding or developing the schools’ practice concerning physical activity. |
| --- |
| 6. Students are involved in deciding or developing the schools’ practice concerning physical activity. |
| 7. The school collaborates with external stakeholders on individual-targeted initiatives concerning physical activity. |
| 8. The school collaborates with external stakeholders on other than individual-targeted initiatives regarding physical activity. |

**Response scale for items no. 5-8:**

| Very high extent  (5) | High extent  (4) | Neutral  (3) | Low extent  (2) | Not at all  (1) | Don’t know  (0) |
| --- | --- | --- | --- | --- | --- |

| SUBFACTOR 3: SCHOOL PRACTICE |
| --- |

| 9. How often do you work with organized or planned activities within physical activity during the school day? | | | | | | |
| --- | --- | --- | --- | --- | --- | --- |
| 10. How often do you have informal conversations with students about physical activity during the school day?  Response scale for items no. 9-10: | | | | | | |
| Daily or multiple times a day  (6) | Almost daily/several times a week  (5) | About once week  (4) | About one to three times a month  (3) | Less frequently  (2) | Never  (1) | Don’t know  (0) |

| SUBFACTOR 4: QUALITY OF DELIVERY |
| --- |

| 11. I have sufficient knowledge and skills to work with physical activity. |
| --- |
| 12. To what extent are you motivated to work with physical activity? |

| **13.** To what extent is your practice concerning physical activity based on the best available knowledge (e.g., recommendations from the national health authority or the local municipality)? |
| --- |
| **14.** To what extent is your practice concerning physical activity based on local knowledge and needs (e.g., requests from employees, student councils, or data from student surveys)? |
| **15.** To what extent do you encourage students to be physically active during leisure time (e.g., by promoting local sports clubs)? |
| **16.** To what extent do you encourage students to use active transportation to and from school (e.g., bicycle)?  **Response scale for items no. 11-16:**   \| Very high extent  (5) \| High extent  (4) \| Neutral  (3) \| Low extent  (2) \| Not at all  (1) \| Don’t know  (0) \| \| --- \| --- \| --- \| --- \| --- \| --- \| |

| SUBFACTOR 5: PHYSICAL AND FINANCIAL |
| --- |

| 17. The school’s physical environment (e.g., outdoor areas, premises) is suitable for working with physical activity. | | | | | |
| --- | --- | --- | --- | --- | --- |
| 18. The school has the financial resources needed for working with student’s physical activity (e.g., to start up new initiatives). | | | | | |
| 19. The management prioritizes competence development concerning physical activity (e.g., by allowing employees to participate in courses). | | | | | |
| 20. I have sufficient time to work with students’ physical activity.  Response scale for items no. 17-20:   \| Very high extent  (5) \| High extent  (4) \| Neutral  (3) \| Low extent  (2) \| Not at all  (1) \| Don’t know  (0) \| \| --- \| --- \| --- \| --- \| --- \| --- \| | | | | | |
|  |  |  |  |  |  |

| SUBFACTOR 6: SCHOOL HEALTH SERVICES |
| --- |

| 21. Do you know who you can refer students to if they want to be more physically active (e.g., a person connected to the school or a person outside of the school)? |
| --- |

**Response scale for item no. 21:**

| Very high extent  (5) | High extent  (4) | Neutral  (3) | Low extent  (2) | Not at all  (1) | Don’t know  (0) |
| --- | --- | --- | --- | --- | --- |

**Whole School approach to Physical Activity (WSA-PA) Questionnaire. Danish Version.**

| SUBFAKTOR 1: KERNEVÆRDIER |
| --- |

| \| 1. Det er en del af skolens kerneværdier at fremme elevernes motion og bevægelse. \| \| \| \| \| \| \| --- \| --- \| --- \| --- \| --- \| --- \| \| 2. Det er skolens ansvar at bidrage til at fremme elevernes motion og bevægelse. \| \| \| \| \| \| \| 3. Skolens ansatte arbejder ud fra en forståelse af, at der er en sammenhæng mellem elevernes motion og bevægelse og læring. \| \| \| \| \| \| \| 4. Ledelsen sætter mål og retning for skolens arbejde inden for motion og bevægelse. \| \| \| \| \| \| \| Svarkategorier til spørgsmål nr. 1-4: \| \| \| \| \| \| \| I meget høj grad  (5) \| I høj grad  (4) \| Hverken i høj eller lav grad  (3) \| I lav grad  (2) \| Slet ikke  (1) \| Ved ikke  (0) \| \|  \|  \|  \|  \|  \|  \| |
| --- | --- | --- | --- | --- | --- | --- | --- | --- | --- | --- | --- | --- | --- | --- | --- | --- | --- | --- | --- | --- | --- | --- | --- | --- | --- | --- | --- | --- | --- | --- | --- | --- | --- | --- | --- | --- | --- | --- | --- | --- | --- | --- |

| SUBFAKTOR 2: SAMARBEJDE OG INVOLVERING |
| --- |

| 5. Bliver skolens ansatte involveret i skolens arbejde med at beslutte eller udvikle skolens praksis inden for motion og bevægelse?? | | | | | |
| --- | --- | --- | --- | --- | --- |
| 6. Bliver skolens elever involveret i skolens arbejde med at beslutte eller udvikle skolens praksis inden for motion og bevægelse??? | | | | | |
| 7. Samarbejder skolen med eksterne aktører om individrettede indsatser inden for motion og bevægelse? | | | | | |
| 8. Samarbejder skolen med eksterne aktører om andet end individrettede indsatser inden for motion og bevægelse? Tænk her på, om I samarbejder med eksterne om udvikling af sundhedspolitikker, kompetenceudvikling af skolens ansatte eller om kollektivt orienterede indsatser, dvs. indsatser målrettet grupper af elever. Det kan fx være, hvis eksterne hjælper skolen med at afholde events, temadage eller undervisningsaktiviteter. | | | | | |
| Svarkategorier til spørgsmål nr. 5-8: | | | | | |
| I meget høj grad  (5) | I høj grad  (4) | Hverken i høj eller lav grad  (3) | I lav grad  (2) | Slet ikke  (1) | Ved ikke  (0) |

| SUBFAKTOR 3: SKOLEPRAKSIS |
| --- |

| 9. Hvor ofte arbejder du med organiserede og planlagte aktiviteter inden for motion og bevægelse i løbet af skoledagen? Tænk både på praktiske eller teoretiske undervisningsaktiviteter, aktiviteter i pauser og i forbindelse med særlige begivenheder (fx events eller temadage). Det kan være noget du selv gør eller i samarbejde med eksterne aktører. | | | | | | |
| --- | --- | --- | --- | --- | --- | --- |
| 10. Hvor ofte har du uformelle samtaler med elever om motion og bevægelse i løbet af skoledagen? | | | | | | |
| Svarkategorier til spørgsmål nr. 9-10: | | | | | | |
| Dagligt eller flere gange dagligt  (6) | Næsten dagligt/flere gange om ugen  (5) | Ca. en gang om ugen  (4) | Ca. en-tre gange om måneden  (3) | Sjældnere (2) | Aldrig  (1) | Ved ikke  (0) |

| SUBFAKTOR 4: KVALITET I PRAKSIS |
| --- |

| 11. Jeg har tilstrækkelig viden og kompetencer til at arbejde med motion og bevægelse. |
| --- |
| 12. Er du motiveret for at arbejde med tiltag inden for motion og bevægelse? |

| **13.** Vurderer du, at din praksis inden for motion og bevægelse er baseret på den bedst tilgængelige viden (det kan fx være anbefalinger fra Sundhedsstyrelsen eller kommunen)? |
| --- |

| **14.** Vurderer du, at din praksis inden for motion og bevægelse er baseret på lokal viden og behov (det kan fx være ønsker fra medarbejdere, elevråd eller data fra trivselsmålinger)? |
| --- |

| **15.** Opfordrer du elever til at dyrke motion og bevægelse i fritiden (fx gennem promovering af lokale idrætsforeninger)? |
| --- |

| **16.** Opfordrer du elever til at anvende aktiv transport til og fra skolen (fx cykling)? |
| --- |

| Svarkategorier til spørgsmål nr. 11-16: | | | | | |
| --- | --- | --- | --- | --- | --- |
| I meget høj grad  (5) | I høj grad  (4) | Hverken i høj eller lav grad  (3) | I lav grad  (2) | Slet ikke  (1) | Ved ikke  (0) |

| \| SUBFAKTOR 5: FYSISK MILJØ OG RESSOURCER \| \| --- \|  \| 17. Skolens fysiske rammer er egnede til at arbejde med motion og bevægelse (fx udendørsarealer, lokaler). \| \| --- \| \| 18. Skolen har et økonomisk råderum, der gør det muligt at arbejde med elevernes motion og bevægelse (fx igangsætte nye tiltag). \| \| 19. Ledelsen prioriterer kompetenceudvikling inden for motion og bevægelse (fx ved at ansatte får mulighed for at deltage i kurser). \| \| 20. Jeg har tilstrækkelig tid til at arbejde med motion og bevægelse. \| | | | | | |
| --- | --- | --- | --- | --- | --- | --- | --- | --- | --- | --- |
| Svarkategorier til spørgsmål nr. 17-20: | | | | | |
| I meget høj grad  (5) | I høj grad  (4) | Hverken i høj eller lav grad  (3) | I lav grad  (2) | Slet ikke  (1) | Ved ikke  (0) |

| SUBFAKTOR 6: SUNDHEDSFAGLIGT PERSONALE |
| --- |

| 21. Ved du, hvem du kan henvise elever til, hvis de gerne vil være mere fysisk aktive? Det kan både være en person på skolen eller en person/organisation udenfor skolen. |
| --- |

**Svarkategorier til spørgsmål nr. 21:**

| I meget høj grad  (5) | I høj grad  (4) | Hverken i høj eller lav grad  (3) | I lav grad  (2) | Slet ikke  (1) | Ved ikke  (0) |
| --- | --- | --- | --- | --- | --- |

**Appendix 4: Factor Structure Diagrams**

**Figure 1S: Factor structure of the Health Promoting School scale – results from the first-order factor analysis**


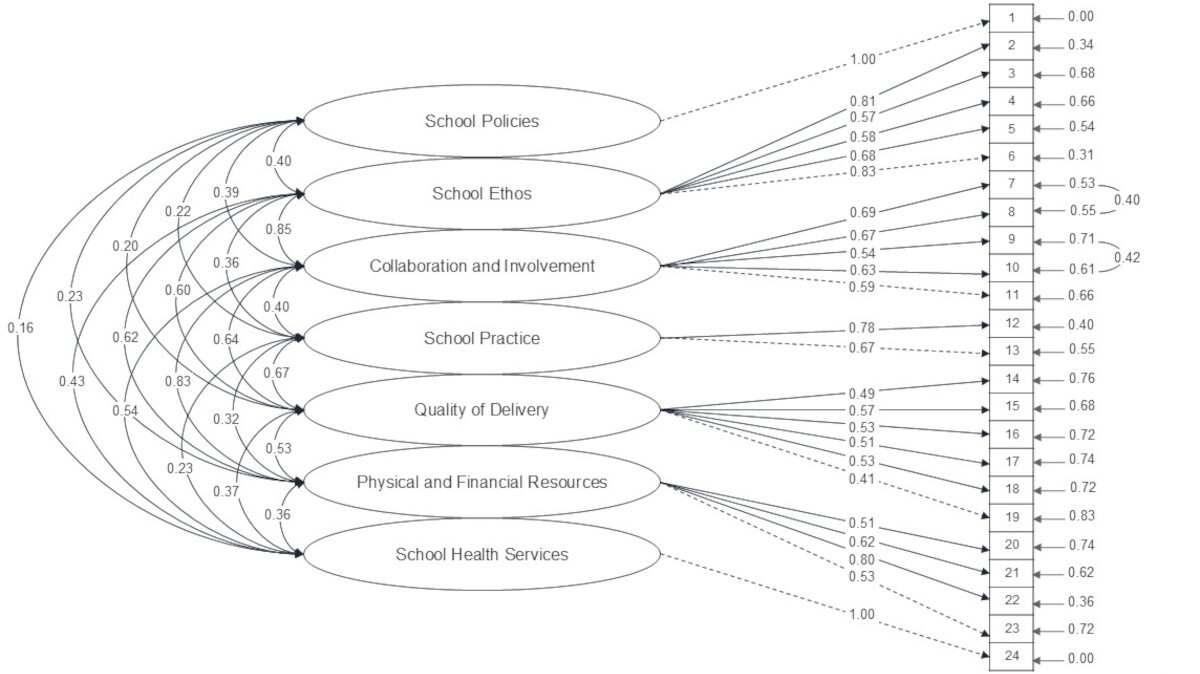


**
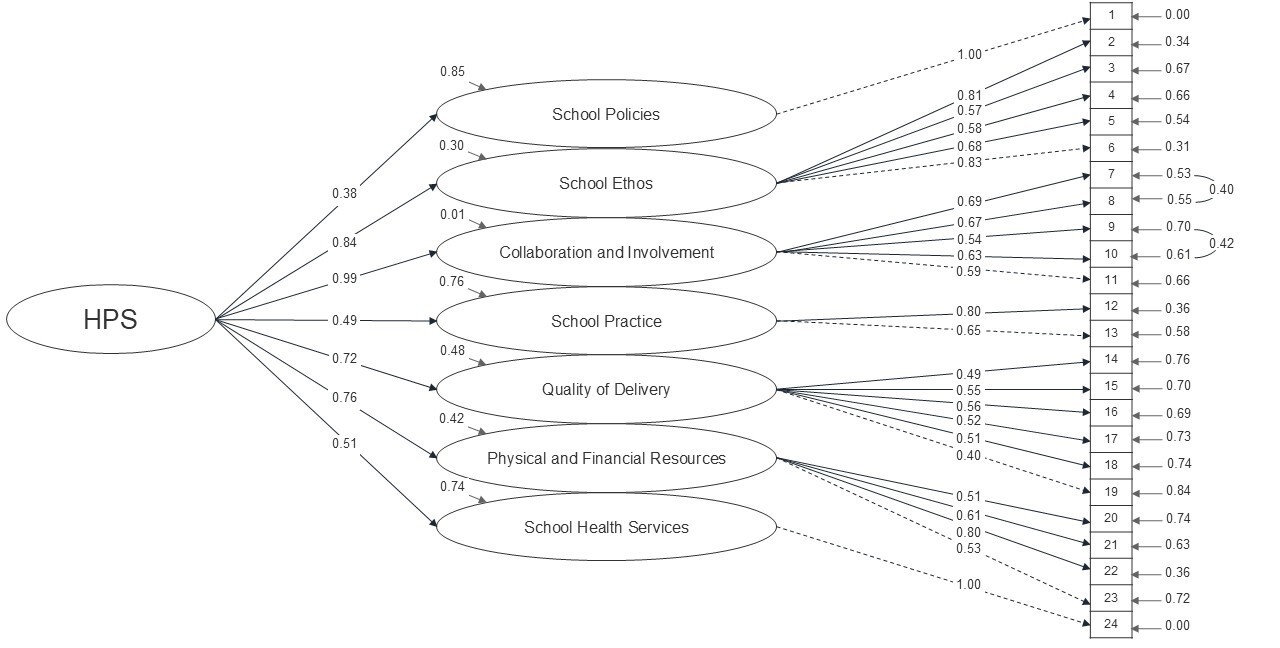
Figure 2S: Factor structure of the Health Promoting School (HPS) scale – results from the second-order factor analysis**

**Figure 3S: Factor structure of the Whole School approach to Physical Activity WSA-PAscale – results from the first-order factor analysis**


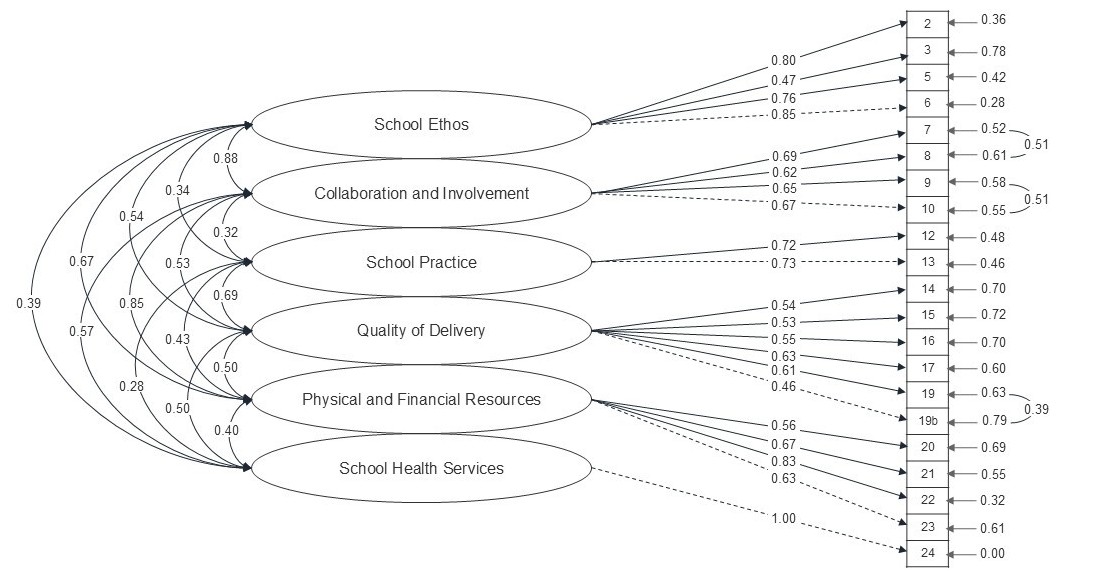


**Figure 4S: Factor structure of the Whole School approach to Physical Activity (WSA-PA) scale – results from the second-order factor analysis**


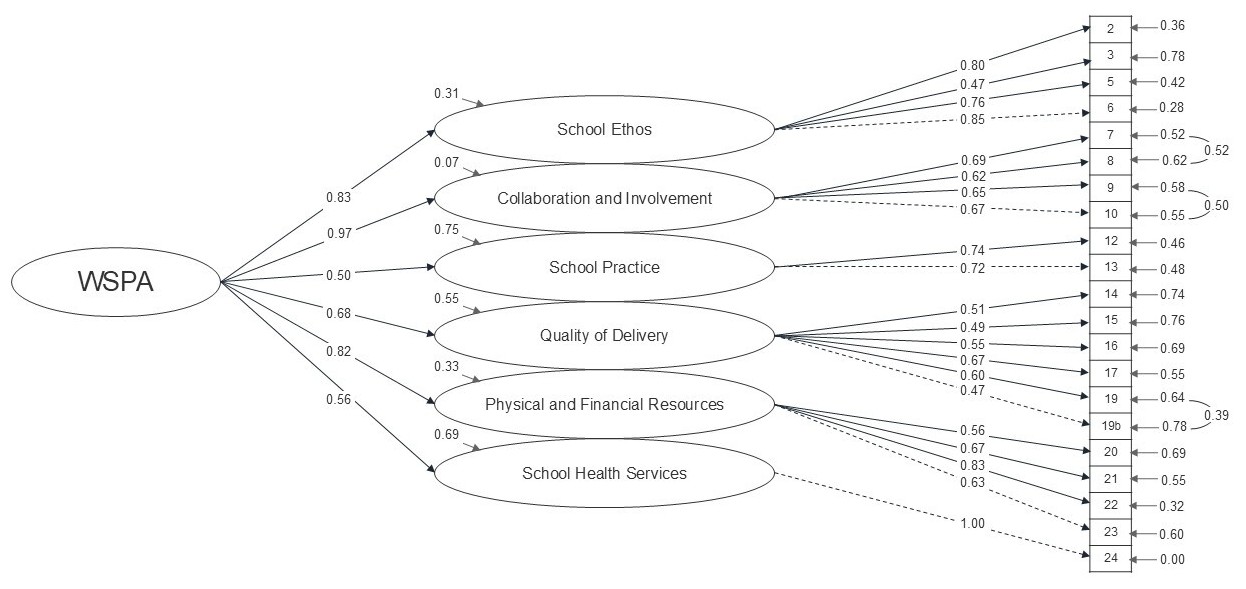


**Appendix 5: Multi-Trait Multi-Methods Results**

**Table SX**

Goodness of fit indices and model comparisons based on the Multi-Trait Multi-Methods (MTMM) analysis

| Goodness of fit indices for the four models based on MTMM analysis | | | | | |
| --- | --- | --- | --- | --- | --- |
|  | Model 1: Correlated Traits/  Correlated Methods | | Model 2: Correlated Methods  (no traits) | Model 3: Perfectly Correlated Traits/Freely Correlated Methods | Model 4: Freely Correlated Traits/Uncorrelated Methods model |
| Root Mean Error of Approximation | | 0.078 | 0.109 | 0.096 | 0.083 |
| Standardized Root Mean Square Residual | | 0.061 | 0.105 | 0.069 | 0.070 |
| Comparative Fit Index | | 0.778 | 0.534 | 0.662 | 0.752 |
| Tucker-Lewis Index | | 0.752 | 0.509 | 0.625 | 0.722 |
| Chi-Square (df)  P-value | | 2376 (766)  0.001 | 4222 (818)  0.001 | 3144 (776)  0.001 | 2577 (767)  0.001 |
| Model comparisons based on ANOVA analysis | | | | | |
| Chi-square diff  P-value | | Ref. | 1846  0.001 | 868  0.001 | 201  0.001 |
